# Nutrient Limitation Governs *Staphylococcus aureus* Metabolism and Niche Adaptation in the Human Nose

**DOI:** 10.1371/journal.ppat.1003862

**Published:** 2014-01-16

**Authors:** Bernhard Krismer, Manuel Liebeke, Daniela Janek, Mulugeta Nega, Maren Rautenberg, Gabriele Hornig, Clemens Unger, Christopher Weidenmaier, Michael Lalk, Andreas Peschel

**Affiliations:** 1 Interfaculty Institute of Microbiology and Infection Medicine, Cellular and Molecular Microbiology, Eberhard-Karls-University Tübingen, Tübingen, Germany; 2 German Center for Infection Research (DZIF), partner site Tübingen, Tübingen, Germany; 3 Institute of Pharmacy, Ernst-Moritz-Arndt University of Greifswald, Greifswald, Germany; 4 Interfaculty Institute of Microbiology and Infection Medicine, Microbial Genetics, Eberhard-Karls-University Tübingen, Tübingen, Germany; 5 Institute for Tropical Medicine, University of Tübingen, Tübingen, Germany; Harvard Medical School, United States of America

## Abstract

Colonization of the human nose by *Staphylococcus aureus* in one-third of the population represents a major risk factor for invasive infections. The basis for adaptation of *S. aureus* to this specific habitat and reasons for the human predisposition to become colonized have remained largely unknown. Human nasal secretions were analyzed by metabolomics and found to contain potential nutrients in rather low amounts. No significant differences were found between *S. aureus* carriers and non-carriers, indicating that carriage is not associated with individual differences in nutrient supply. A synthetic nasal medium (SNM3) was composed based on the metabolomics data that permits consistent growth of *S. aureus* isolates. Key genes were expressed in SNM3 in a similar way as in the human nose, indicating that SNM3 represents a suitable surrogate environment for *in vitro* simulation studies. While the majority of *S. aureus* strains grew well in SNM3, most of the tested coagulase-negative staphylococci (CoNS) had major problems to multiply in SNM3 supporting the notion that CoNS are less well adapted to the nose and colonize preferentially the human skin. Global gene expression analysis revealed that, during growth in SNM3, *S. aureus* depends heavily on *de novo* synthesis of methionine. Accordingly, the methionine-biosynthesis enzyme cysteine-γ-synthase (MetI) was indispensable for growth in SNM3, and the MetI inhibitor DL-propargylglycine inhibited *S. aureus* growth in SNM3 but not in the presence of methionine. Of note, *metI* was strongly up-regulated by *S. aureus* in human noses, and *metI* mutants were strongly abrogated in their capacity to colonize the noses of cotton rats. These findings indicate that the methionine biosynthetic pathway may include promising antimicrobial targets that have previously remained unrecognized. Hence, exploring the environmental conditions facultative pathogens are exposed to during colonization can be useful for understanding niche adaptation and identifying targets for new antimicrobial strategies.

## Author Summary

Many severe bacterial infections are caused by endogenous pathogens colonizing human body surfaces. Eradication of notorious pathogens such as *Staphylococcus aureus* from risk patients has become an important preventive strategy. However, efficient decolonization agents are rare, and the living conditions of colonizing pathogens have hardly been studied. Using a combined metabolomics and transcriptomics approach, we explored the metabolism of *S. aureus* during colonization of its preferred niche, the human nose. Based on nasal metabolite profiles, a synthetic nasal medium (SNM3) was composed, enabling steady growth of *S. aureus* but not of staphylococcal species preferentially colonizing the human skin. Marker gene expression was similar in SNM3 and the human nose, and genome-wide expression analysis revealed that amino acid biosynthesis, in particular that of methionine, is critical for *S. aureus* during colonization. An inhibitor of methionine biosynthesis had anti-staphylococcal activity in SNM3 but not in complex media, and transcription of the *S. aureus* target enzyme was strongly up-regulated in human noses. Furthermore, mutants defective in methionine biosynthesis exhibited strongly compromised nasal colonisation capacities in a cotton rat model. Altogether, our results indicate that the elucidation of *in vivo* metabolism of pathogens may lead to the identification of new antimicrobial targets and compounds.

## Introduction

*Staphylococcus aureus* is a major cause of human invasive infections ranging from superficial skin and soft tissue infections to severe disseminated diseases such as sepsis and endocarditis [1]. *S. aureus* is also a human commensal and part of the microbiota in healthy individuals, which facilitates its access to sterile tissues via open wounds and catheter entry sites. *S. aureus* can be isolated from various human body surfaces such as the pharynx, axillae and perineum but its main ecological niche and reservoir is known for long to be the human nose [2]–[4]. In contrast, coagulase-negative staphylococci (CoNS), such as *Staphylococcus epidermidis*, have a much lower virulence potential and use different areas of the skin as their major habitats [5]. The basis of staphylococcal host and niche-specificity has remained unknown.

Analysis of nasal carriage over long time periods has identified three types of *S. aureus* carriers [6]. About 20% of the human population can be regarded as *non-carriers*, who are never or only in very rare instances colonized with low bacterial numbers. In contrast, *intermittent carriers* show alternating periods of non-carrier status and colonisation by various *S. aureus* strains. The number of bacteria per isolation can be highly variable. The third group of roughly 20% *persistent carriers* is characterised by the presence of *S. aureus* in nearly all nasal swabs, usually at high bacterial numbers and with one specific strain per person over time. Recently, it has been suggested to distinguish only between carriers and non-carriers because of similar *S. aureus* nasal elimination kinetics and anti-staphylococcal antibody profiles in intermittent- and non-carriers [7].

Recent studies have shown that being an *S. aureus* carrier bears a higher risk of invasive S. *aureus* infections, predominantly by the carriers' own strain [8], but a lower risk of infection-associated mortality compared to being a non-carrier [9]. The reasons for the underlying predisposition, which may involve individual differences in epithelial ligands for bacterial adhesins, local host defense, or availability of nutrients in the nose, have remained unclear. While some polymorphisms in immunity-related genes are weakly associated with the carrier status, the human predisposition appears to have multifactorial reasons [10]. On the bacterial side several factors required for nasal colonization have been identified. Wall teichoic acid (WTA) polymers at the staphylococcal surface [11], [12] and cell-wall anchored proteins, such as ClfB [13], [14] and IsdA [15], have been found to be required for nasal colonization and adhesion to nasal epithelial cells. While the epithelial receptor for WTA is still unknown, ClfB and IsdA bind to cytokeratin 10 and loricrin, major components of human squamous epithelial cells, and IsdA also binds to the matrix protein involucrin [15], [16]. Recently, up-regulation of WTA-biosynthetic genes *tagO* and *tarK* and of *clfB* and *isdA* during nasal colonisation has been shown in nasal swab samples from human volunteers [17] and in the cotton rat model of *S. aureus* nasal colonisation [18], underscoring the importance of these factors in nasal colonization. *S. aureus* encounters iron-limiting conditions in the nose because *isdA* expression is strictly dependent on iron limitation [19], and haemoglobin has recently been shown to promote *S. aureus* nasal colonization [20].

The human body can be regarded as a chemostat, where the nutrients required for bacterial growth are replenished over time and allow growth of bacteria within human microenvironments [21]. While several *S. aureus* nasal adhesion factors have been studied in the past, nothing is known about the growth conditions in nasal fluid such as the availability of carbon and nitrogen sources and the metabolic activities of *S. aureus* in its nasal habitat. Knowledge of metabolite availabilities and utilisation patterns could direct the identification of important metabolic enzymes that are essential during infection or colonization by *S. aureus* and could serve as targets for new antibiotics. Along this line enzymes from the folic acid biosynthetic pathway or the isoleucyl-tRNA synthetase are valuable targets for widely used anti-staphylococcal antibiotics such as cotrimoxazole or mupirocin, respectively. Mupirocin is frequently used to eliminate *S. aureus* from the noses of high-risk patients [22], but the increasing resistance to mupirocin [23] and almost all antibiotics used to prevent or treat staphylococcal infections puts urgency to the development of new antimicrobial compounds. For this purpose the most critical and ‘drugable’ metabolic pathways of *S. aureus* need to be identified.

In this study we elucidated the abundance of potential nutrients for *S. aureus* in the human nose by metabolomics analysis of nasal secretions. We found a high diversity but low concentrations of metabolites and no significant differences in the composition of secretions from *S. aureus* carriers and non-carriers. A synthetic nasal medium (SNM3) was composed based on the metabolomics data. *S. aureus* growth in SNM3 led to similar gene expression patterns as during *in vivo* colonization. While *S. aureus* isolates grew steadily in SNM3, CoNS did not, indicating that *S. aureus* is particularly well adapted to life in the human nose. Analysis of global gene expression in SNM3 revealed that the methionine-biosynthetic pathway may be a critical target for new anti-colonization drugs. In support of this notion an inhibitor of methionine biosynthesis had antimicrobial activity against *S. aureus* in SNM3 but not in complex media. The inhibitor's staphylococcal target gene was strongly up-regulated during human nasal colonization, and deletion of the target gene led to reduced colonisation ability of the respective mutant in the cotton rat colonisation model. Thus, deciphering the *in vivo* metabolism of pathogens represents a valuable strategy for defining new antimicrobial targets.

## Results

### Human nasal secretions have similar composition in *S. aureus* carriers and non-carriers

**Figure 1:**
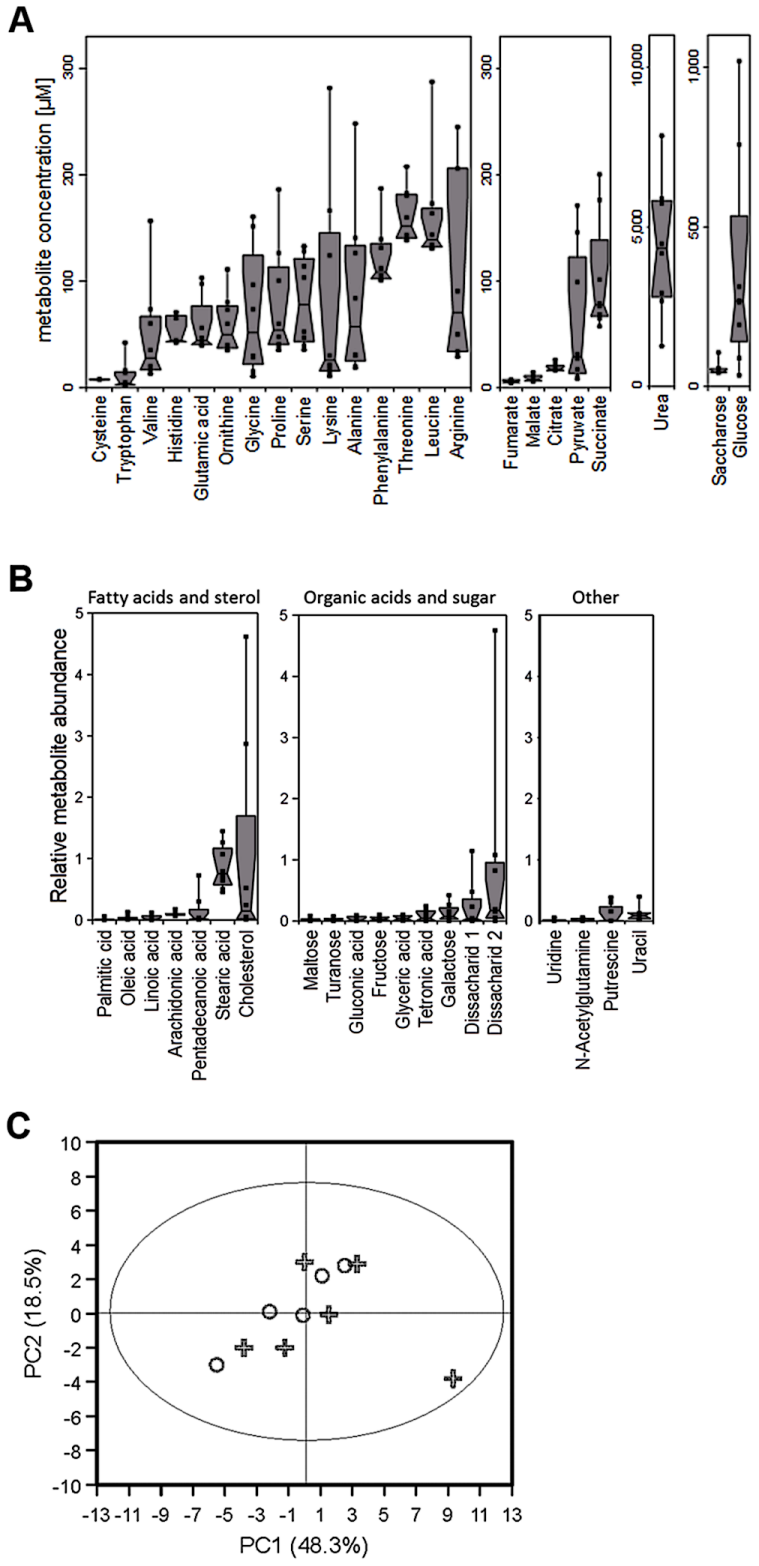
Metabolite composition of nasal secretions in *S. aureus* carriers and non-carriers. (A, B) Metabolite quantification by GC-MS from eight sample donors, micromolar concentrations are given for compounds with standard dilutions measured (A), for other substances relative abundances are given against internal standards (B). Upper and lower box limits and the horizontal lines within the boxes represent 25 and 75% percentiles and the medians, respectively. The whiskers of the plots indicate minimum and maximum range, additionally showing the data points. (C) Differences in metabolite profiles in nasal secretions from six carriers (crosses) and seven non-carriers (circles) analyzed by principal component analysis of all metabolite levels. Principal component 1 vs. 2 is shown, explaining in total 67.9% of the PCA model. Metabolite values were log-transformed and scaled by unit variance.

The abundance of potential nutrients in nasal secretions has never been described. In order to explore the metabolic lifestyle of *S. aureus* during nasal colonization, the amounts of small organic compounds in secretions from eight volunteers who were not carriers of *S. aureus*, were analyzed by metabolomics (Fig. 1A, Table 1). Similar amounts of amino acids and organic acids were found in the micromolar range in the eight samples. Urea was by far the most abundant organic substance at concentrations of 2.5–7.5 mM. Glucose exhibited the highest concentrations among carbohydrates with large variation between 35 µM and ca. 1 mM, while only very low amounts of other mono- or disaccharides were detected. Most of the proteinogenic amino acids and ornithine were present at average concentrations between 50 and 150 µM, while tryptophan and cysteine were detected only at very low concentrations around 10 µM. Some amino acids were not found (methionine, glutamine, tyrosine, isoleucine, asparagine, and aspartate). Whereas the carboxylic acids fumarate, malate and citrate were detected at about 5–25 µM, pyruvate and succinate were usually present at much higher concentrations (Table 1). No lactate and only trace amounts of several other substances, including fatty acids, cholesterol and pyrimidines, were found (Fig. 1B).

**Table 1:** Concentration ranges of metabolites and inorganic ions in human nasal secretions and amounts of these compounds in the chemically defined medium SNM.

Compound	Conc. Range *in vivo* [µM]	Mean value ± SEM [µM]	Concentration in SNM [µM]
Alanine	19.0–248.0	87.5±28.6	150
Arginine	29.1–244.9	108.0±38.9	100
Cysteine	7.3–8.58	7.79±0.17	10
Glutamic acid	39.8–103.4	58.3±9.4	100
Glycine	10.8–160.5	71.0±21.3	150
Histidine	42.4–70.9	52.9±4.65	50
Leucin	130.5–287.2	162.2±18.7	300
Lysine	11.1–281.3	83.4±35	150
Ornithine	35.2–111.3	59.4±9.68	100
Phenylalanine	101.0–187.1	122.6±10.6	150
Proline	35.5–186.3	79.8±19.1	150
Serine	35.7–132.9	81.7±14.8	120
Threonine	138.5–207.8	161.8±9.13	200
Tryptophane	2.1–42.3	11.0±4.88	20
Valine	13.1–156.5	49.1±17.3	100
**Organic acids**			
Citric acid	16.2–26.3	18.9±1.27	20
Fumaric acid	4.6–7.7	6.0±0.46	5
Maleic acid	6.3–14.5	8.9±1.14	10
Pyruvic acid	8,6–170.9	63.9±23.1	100
Succinic acid	57.9–200.4	103.2±19.3	200
**Glucose**	35.9–1018.1	367.4±120.8	700
**Urea**	1263.0–7846.9	4374.3±742.6	5000
**Inorganic Ions** a		**[mM] ± SEM**	**[mM]**
Sodium		127±6	130
Chloride		140±7	145
Potassium		27±3	25
Phosphate		10	10
Magnesium		0.5	0.5
Sulphate		0.5	0.5
Calcium^b^		5	0

The concentrations of additional SNM components are given in Table 2.

^a^ Concentrations are given as published for sodium, chloride, potassium and calcium in [24] and for phosphate, magnesium and sulphate in [25]. No SEM were available for phosphate, magnesium, sulphate, and calcium.

^b^ Calcium was omitted from SNM because it led to precipitates.

To confirm the observed results and to investigate potential differences between *S. aureus* carriers and non-carriers, the metabolite composition in nasal secretions from six *S. aureus* non-carriers were compared with those from seven *S. aureus* carriers. The metabolite patterns of carriers exhibited a similar degree of variation as in non-carriers but no significant difference between the two groups of donors for any of the detected compounds (Fig. 1C). Thus, the human *S. aureus* carrier status is not associated with a notable difference in nasal nutrient supply, and the metabolic activities of *S. aureus* do not seem to have a major impact on the overall metabolite concentrations in the nose.

### Synthetic nasal medium (SNM) permits steady growth of *S. aureus*

The average nutrient concentrations found in human nasal secretions were used to compose a synthetic medium for simulating *S. aureus* growth in the nose (Table 1). Amino acids and glucose were added to SNM at the upper limit of the detected concentration ranges found in the samples but not at higher amounts than twice the mean concentration values. The amounts of inorganic ions in nasal secretions have previously been described [24], and these values served as a basis for the salt content of SNM. The synthetic medium was buffered with 10 mM phosphate buffer (pH 7.2), which corresponds to the previously described nasal phosphate content [25]. The concentrations of essential cofactors such as vitamins and trace elements in nasal secretions were below detection limits. Since earlier studies had shown that *S. aureus* requires minimal amounts of these compounds [26], highly diluted standard vitamin and trace element solutions were included in SNM. Moreover, because recent gene expression data have shown that *S. aureus* encounters iron-limited conditions in the human nose [17], iron was omitted from the trace element solution, and SNM was supplemented with 200 µM of the iron-complexing agent 2, 2′-bipyridin, which has been shown to confer iron limitation in *S. aureus* [27]. The concentrations of inorganic salts, 2, 2′-bipyridine, trace elements, and cofactors in SNM are listed in Table 2.

**Table 2:** Inorganic salt, 2, 2′-bipyridine, trace element, and cofactor concentrations in SNM.

Salts, buffer^a^, bipyridine	Final concentration [mM]
Sodium phosphate buffer (pH 7.2)	5
Potassium phosphate buffer (pH 7.2)	5
NaCl	125
KCl	20
MgSO_4_	0.5
2, 2′-Bipyridine	0.2
**Trace elements** b	**[µg/l]**
ZnCl_2_	70
MnCl_2_	100
H_3_BO_3_	6
CoCl_2_	190
CuCl_2_	2
NiCl_2_	24
MoNa_2_O_4_	36
**Cofactors** b	**[µg/l]**
Cyanocobalamine	100
p-Aminobenzoic acid	80
Biotin	20
Nicotinic acid	200
Ca-D-Panthothenic acid	100
Pyridoxamine-2HCl	300
Thiamine-dichloride	200
Riboflavin	200

Final concentrations of organic compounds that could be detected by metabolomics are given in Table 1.

^a^ Concentrations derived from previously published data from human nasal secretions [24], [25].

^b^ standard trace element and cofactor solutions.

**Figure 2:**
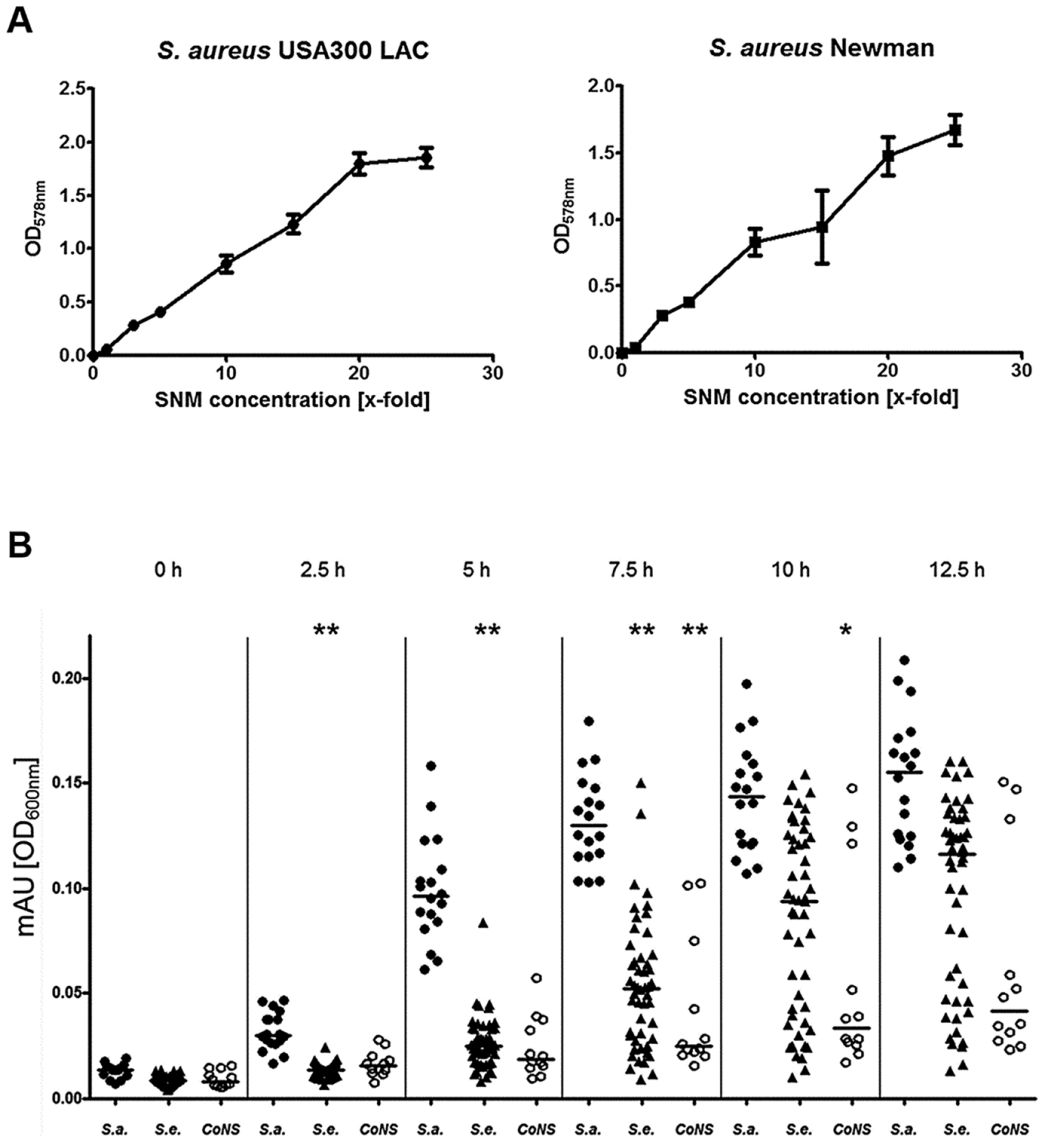
Capacities of *S. aureus* and CoNS strains to grow in synthetic nasal medium (SNM). (A), growth of *S. aureus* USA300 LAC and *S. aureus* Newman in SNM with increasing (x-fold) concentrations of amino acids, organic acids and glucose after 90 h growth. The mean values and SEM for three independent cultures of each concentration is shown. (B) Growth of nasal isolates of *S. aureus* (filled circles), *S. epidermidis* (triangles) and other coagulase-negative staphylococci (CoNS) (open circles) in liquid SNM3 after the indicated incubation times. The means at each time point are shown as horizontal lines for each group. Statistically significant differences vs. *S. aureus* calculated by the ANOVA Kruskal-Wallis test with Post-test Dunns and 95% confidence intervals are indicated: *, p≤0.05; **, p≤0.01. Cultures were vigorously shaken at 37°C in A and B.

The community-associated methicillin-resistant *S. aureus* (CA-MRSA) clinical isolate USA300 LAC and the laboratory strain Newman were used to evaluate if *S. aureus* is able to grow in SMN. Despite the very low amount of 238 mg amino acids per liter, both strains showed reproducible but moderate growth in SNM. This is in agreement with the rather low bacterial numbers found in swabs from the human nose [28]. In contrast, much higher bacterial densities have been found in microbiomes which are in contact with ingested food e.g. in the human mouth or gut [29], [30]. When we increased the concentration of amino acids, organic acids and glucose in SNM, the maximal bacterial densities also increased until they reached a plateau at approximately 20-fold nutrient concentration for *S. aureus* USA300 (Fig. 2A). Because epithelial secretions are continuously produced and removed, nutrient concentrations should remain more or less constant at the surface of nasal tissues, while they are continuously decreasing in a test tube culture. In agreement with this notion *S. aureus* Newman reached 2.4-fold higher bacterial numbers when grown in SNM in a continuous flow system compared to static SNM (Supplementary Figure S1). Because several of the subsequent experiments involved growth on SNM agar plates or in multiple parallel cultures, which could not be performed in a continuous flow system, we used SNM with threefold increased amino acid, organic acid and glucose concentration (SNM3) in subsequent experiments.

### *S. aureus* is better adapted to growth in SNM3 than CoNS

In order to validate the capacity of SNM3 to simulate living conditions for staphylococci in the human nose, 87 different staphylococcal strains from the anterior nares of 37 human volunteers were isolated, and growth of the corresponding 18 *S. aureus*, 57 *S. epidermidis*, and 12 other CoNS strains (*Staphylococcus capitis*, *Staphylococcus lugdunensis*, *Staphylococcus warneri*, *Staphylococcus hominis*) in SNM3 was compared.

**Figure 3:**
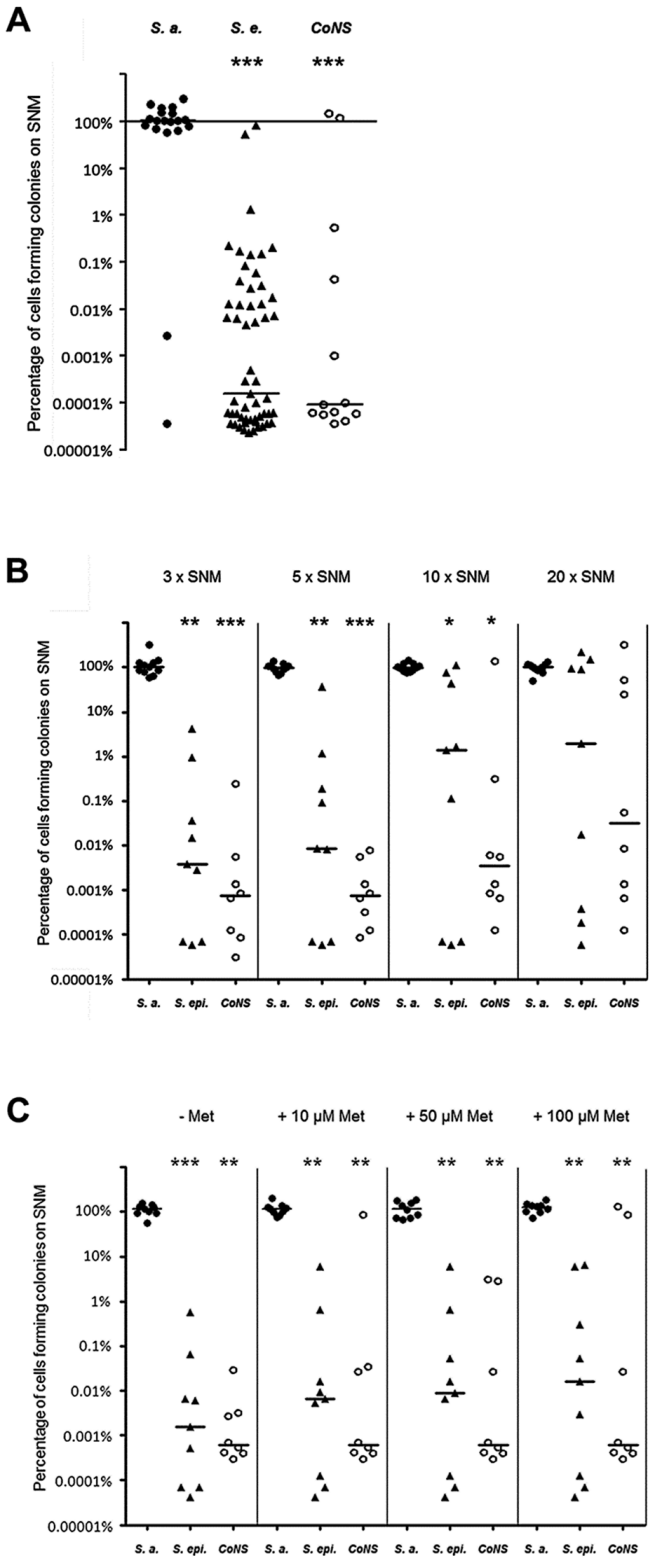
Colony formation abilities on SNM agar of *S. aureus*, *S. epidermidis* and other CoNS strains isolated from human nares. (A) Serial dilutions of all nasal isolates grown in BM overnight were spotted on BM or 3× SNM (SNM3) agar. The y-axis shows the percentage of cells forming colonies on SNM3 compared to BM. The influence of enhanced nutrient concentrations in 5×, 10×, and 20× SNM (B) or of 10–100 µM methionine in SNM3 (C) on the colony-forming ability was analyzed with a subset of strains shown in Figure 3A. *S. aureus* is shown as filled circles, *S. epidermidis* as triangles and other coagulase-negative staphylococci (CoNS) as open circles. The horizontal bar indicates the median of each group of strains. Statistically significant differences calculated by the ANOVA Kruskal-Wallis test with Post-test Dunns and 95% confidence intervals are indicated: *, p≤0.05; **, p≤0.01; ***, p≤0.001.

All *S. aureus* grew in SNM3 liquid cultures without long lag-phases and reached their highest densities after about 12.5 hours (Fig. 2B). When serial dilutions of the same strains were spotted on SNM3 agar, which should correspond better to the sessile lifestyle on the nasal epithelial surface than liquid SNM3, identical CFUs as on complex medium agar (basic medium, BM) were found for all *S. aureus* except for two strains (Fig. 3A). This indicates that SNM3 offers efficient growth conditions for the vast majority of nasal *S. aureus* strains. In contrast, most of the *S. epidermidis* and other CoNS grew in liquid SNM3 only after long lag-phases and often with much longer generation times compared to *S. aureus* (Fig. 2B). Moreover, only a very small percentage of cells (one to ten out of a million viable cells) of the *S. epidermidis* and other CoNS strains were able to form colonies on SNM3 agar (Fig. 3A). This property appeared to be a stable trait because sub-cultivation of such outgrowing clones resulted in much higher numbers of colonies on SNM3 compared to the parental clones (data not shown). Hence, most *S. aureus* appear to be metabolically well adapted to life in the human nose, whereas the vast majority of CoNS exhibited arrested growth with only a small minority of cells starting multiplication on SNM3 agar. These differences reflect recent findings that the nose of permanent *S. aureus* carriers usually contains substantially lower numbers of CoNS than *S. aureus* [7], [31].

To investigate if *S. aureus* is simply better adapted to dilute nutrient concentrations than CoNS, a selection of strains, whose ability to form colonies on SNM3 was in the median range, was tested for colony formation on SNM agar with three, five, ten, and twenty-fold concentrated nutrients. As shown in Figure 3B, concentrating nutrients in SNM agar plates ten to twenty-fold improved the outgrowth of most of the tested *S. epidermidis* and some other CoNS strains, but only some strains reached similar growth capacities as *S. aureus*, while most formed colonies at several magnitudes lower numbers than *S. aureus* even at the highest nutrient concentrations. Thus, CoNS appear to be much less capable of adapting to diluted nutrient concentrations than *S. aureus* and exhibit enormous intra-species variation in their capacities to grow on SNM3 agar, even when nutrient concentrations were strongly increased.

### Expression of *S. aureus* marker genes in SNM3 is similar to that in the nose

**Figure 4:**
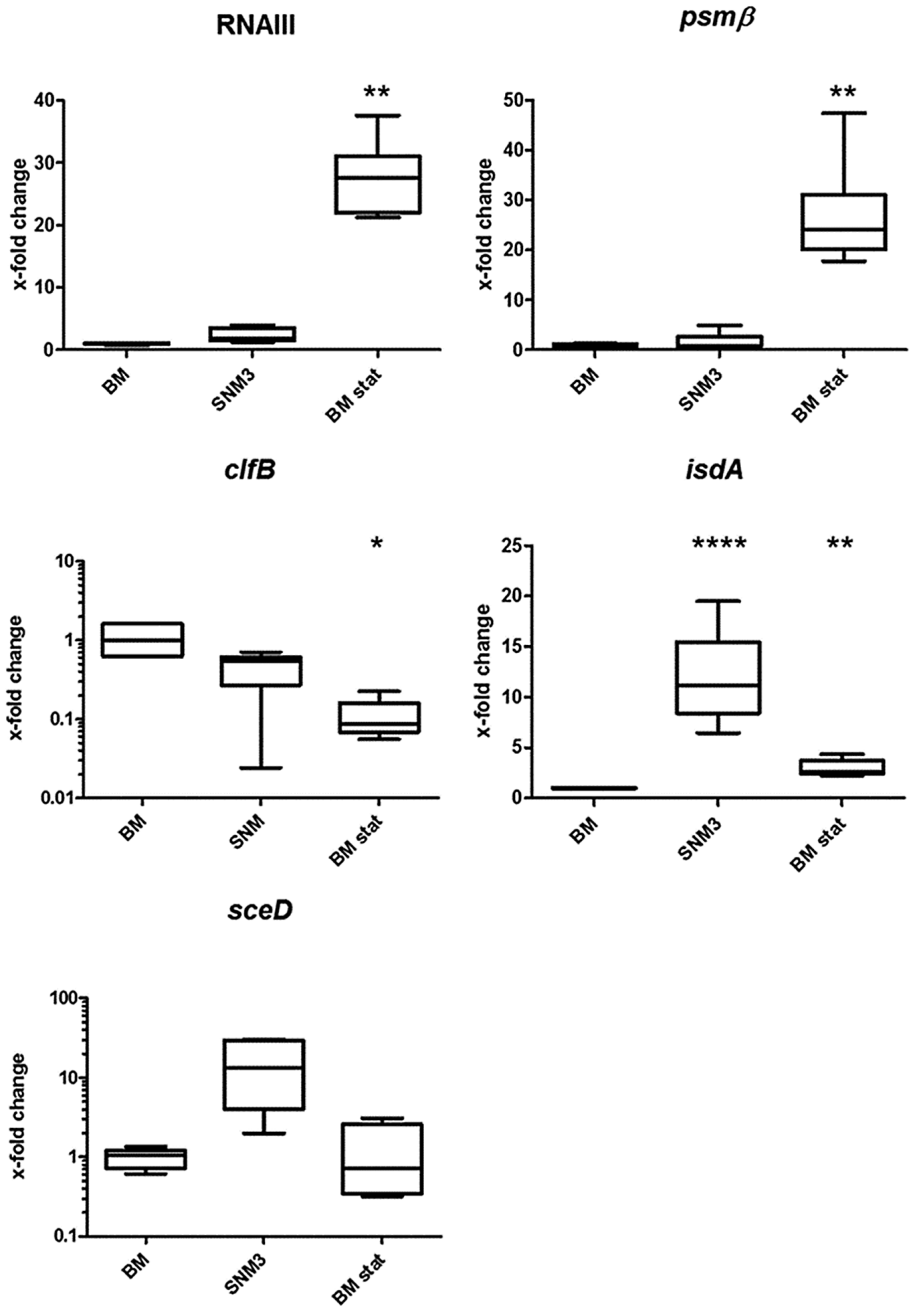
Relative gene expression of selected genes of *S. aureus* USA300 LAC in BM and SNM. Gene expression was assayed by qRT-PCR for at least 6 independent cultures growing in BM or SNM3 or in stationary phase BM cultures (BM stat) each. Analyzed transcripts represent RNAIII, phenol soluble modulins β1 and β2 (*psm*), clumping factor B (*clfB*), iron-regulated surface determinant A (*isdA*), and a lytic transglycosylase (*sceD*). The upper and lower box limits and the horizontal lines within the boxes represent 25 and 75% percentiles and the means, respectively. The whiskers of the plots indicate minimum and maximum values. Statistically significant differences vs. BM calculated by the unpaired, two-tailed Student's *t* test with Welch's correction are indicated: *, p≤0.05; **, p≤0.01; ***, p≤0.001; ****, p≤0.0001.

Two recent studies have described if and how efficiently critical *S. aureus* genes are transcribed during nasal colonization of humans or cotton rats [17], [18]. In order to evaluate if growth in SNM3 leads to similar transcriptional profiles as found during *in vivo* colonization, we compared marker gene expression of *S. aureus* USA300 actively growing in SNM3 or BM by quantitative RT-PCR (qRT-PCR) (Fig. 4). Bacteria grown to stationary phase in BM (BM-stat) were also included because this growth condition has recently been used to assess intranasal expression of relevant *S. aureus* marker genes [17], [18]. The global virulence regulator RNAIII, which responds to the concentration of a secreted *agr* autoinducer peptide (quorum sensing), and the *agr*-controlled *psmß* have been shown to be only moderately expressed in the nose [17], [18], and artificial induction of the RNAIII transcript reduces the nasal colonization capacity of *S. aureus* in the cotton rat model [20]. These previous findings corresponded to the low expression of RNAIII and *psmß* in BM and SNM3 compared to expression in BM stat. Also, the keratin-binding adhesin gene *clfB* was expressed in SNM3 at a similar level as in growing BM cultures, corresponding to the expression profile found recently in human noses [17]. Moreover, expression of the iron-regulated *isdA* and the lytic transglycosylase gene *sceD*, found to be up-regulated in the nose compared to BM or BM-stat cultures [17], [18], was also enhanced in SNM3. Taken together, these data indicate that the composition of SNM3 provides suitable conditions for *in vitro* simulation of *S. aureus* growth and gene expression in the nasal habitat.

### Global *S. aureus* gene expression in SNM3 reveals an important role of amino acid anabolism during nasal colonization

**Figure 5:**
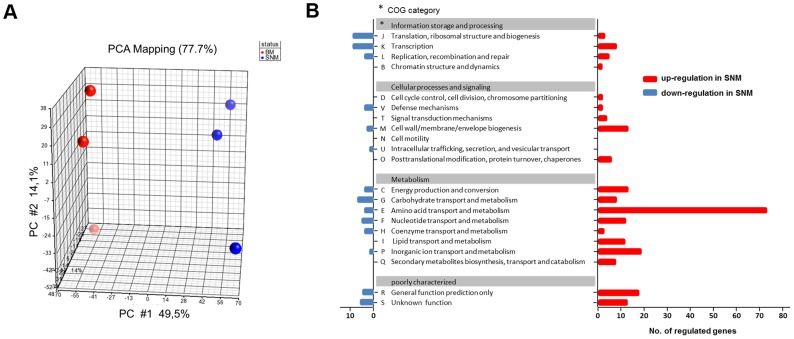
Differences in the transcriptome of *S. aureus* USA300 grown in SNM3 and complex medium (BM). (A) PCA mapping of transcriptome profiles from BM compared to SNM3 cultures from microarray experiments. Each sphere represents an individual GeneChip result with the plotted location based upon the correlation of each sample relative to the others. Results from three independent BM and SNM cultures are compared. (B) Functional classes of genes (COG, Clusters of Orthologous Groups of proteins) detected in the microarray analysis, which were more than two-fold up- (red) or down-regulated (blue) and p<0.05 in SNM3 compared to complex medium (BM) are shown. The x-axis indicates the number of differentially regulated genes in each COG subgroup. The genes of subgroups E, M, and P are listed in detail in the supplementary Figure S2.

SNM3-grown *S. aureus* cultures enabled us to monitor global gene expression under conditions reflecting nasal colonization and to compare these with expression profiles from previous studies, which have usually used *S. aureus* grown in complex media. RNA from *S. aureus* USA300, actively growing either in SNM3 or BM, was hybridized to Affymetrix microarrays and analyzed with respect to basic cellular and metabolic pathways (data deposited under GEO Series accession number GSE43712). Multivariate data analysis was used to show differences or similarities between the transcriptomic data. Principal component analysis (PCA) confirmed that the three biological replicates performed for each of the two conditions led to very reproducible results, with substantial differences in SNM3 or BM-derived transcription profiles and a PCA mapping value of 77.7% (Fig. 5A).

Expression of 521 signals corresponding to 341 genes differed more than two-fold upon growth in SNM3 vs. BM with p-values below 0.05 (Fig. 5B). Thus, complex media represent a very artificial situation for *S. aureus* that differs profoundly from colonization-related conditions. A total of about 12.6% of the genes categorized in the “Clusters of Orthologous Groups of proteins” (COG) database [32] as being involved in “cellular processes and signalling” (Fig. 5B) showed more than two-fold expression differences between the two growth conditions, with 22 genes being up- and only six down-regulated. Most pronounced in this group was the two- to threefold higher expression of various capsule biosynthesis genes (functional category M) in SNM3 compared to BM (Supplementary Fig. S2). This finding is in agreement with the crucial role of the capsule in *S. aureus* nasal colonization recently shown in a rodent model [33]. The up-regulated signals also included the genes for the iron-regulated IsdC surface protein and the osmoprotectant system OpuCC and OpuD. In the group of genes categorized for “information storage and processing” 10.6% of the genes were differentially expressed, 13 genes were up- and 20 down-regulated including various transcriptional regulators.

**Figure 6:**
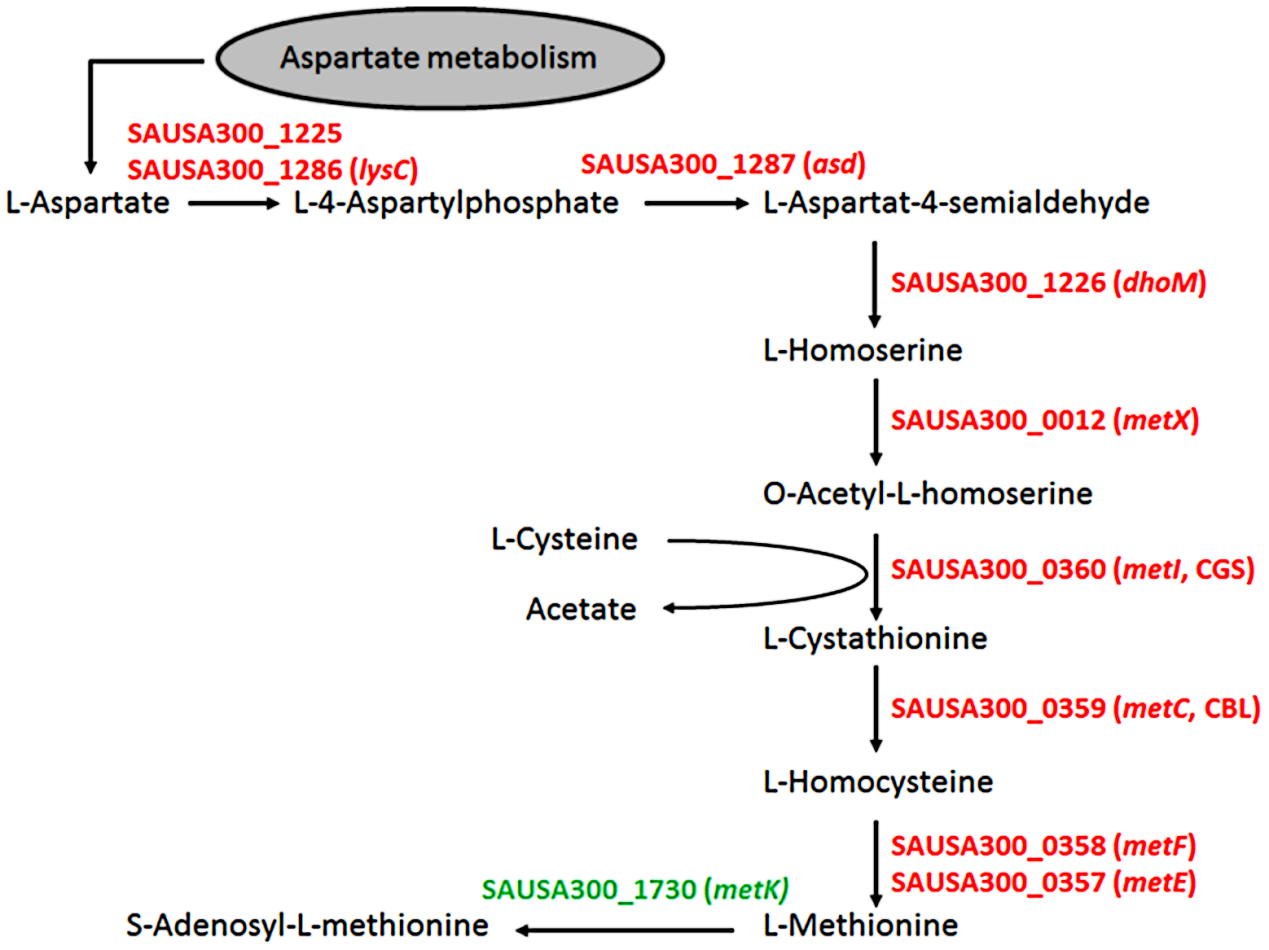
Schematic representation of the methionine-biosynthetic pathway of *S. aureus* USA300 (adapted from KEGG pathway database). All genes shown in red were up-regulated in SNM3 compared to BM as determined by microarray analysis, with SAUSA300_0357 to SAUSA300_0360 exhibiting the strongest effects (13 to 32-fold). The *metK* gene, shown in green, was expressed at similar levels in BM and SNM3. The cystathionine-γ-synthase (MetI) represents the target for DL-propargylglycin.

As expected, most of the major differences were found among genes governing the central “metabolism” (26.6% of all genes assigned to this group), in which 54% of all genes from the functional group E (“amino acid transport and metabolism”) differed more than twofold in expression between the chosen growth conditions. Here, 79 genes were up- and only three down-regulated in SNM3 compared to BM (Supplementary Fig. S2). Significant up-regulation of amino acid biosynthesis genes was observed for glutamate, histidine, lysine, valine, leucine, isoleucine and methionine in SNM3 compared to BM. Remarkably, all of these amino acids, except for methionine and isoleucine, are integral components of SNM3. Whereas isoleucine can easily be generated from threonine, methionine has to be synthesized from aspartate, which was also not detectable in nasal secretions and therefore was not included in SNM3. Via O-acetyl-L-homoserine, aspartate reacts in a transsulfuration reaction with cysteine to form L-cystathionine and L-homocysteine (Fig. 6). The genes for cystathionine-γ-synthase (*metI*, SAUSA300_0360) and cystathionine-β-lyase (*metC*, SAUSA300_0359), whose gene products are responsible for these enzymatic reactions, were 26- to 32-fold up-regulated and exhibited the strongest up-regulation of all genes in SNM3. Among the methionine-biosynthetic genes those for MetE (5-methyltetrahydropteroyltriglutamate-homocysteine S-methyl-transferase) and its homologue MetF SAUSA300_0358 (bifunctional homocysteine S-methyltransferase), responsible for the conversion of L-homocysteine to L-methionine, were 13 to 14-fold up-regulated. The strong up-regulation of two L-methionine ABC-transport systems (SAUSA300_0435-0437 and SAUSA300_0796-0798) underscored the importance of methionine supply during growth in SNM3. In addition to the biosynthetic operons of the above mentioned amino acids, many ABC-type dipeptide/oligopeptide (*opp* genes) as well as metal ion transporters (especially for iron-siderophores), were strongly up-regulated in SNM3 (Supplementary Fig. S2, functional category P). The presence of iron-limiting conditions in SNM3 was reflected by distinct up-regulation of the operon for biosynthesis of staphyloferrin B (also called staphylobactin, *sbnABCDEFGHI*; SAUSA300_0118 to SAUSA300_0126), a potent siderophore facilitating the extraction of iron from human transferrin [34]. The ferric uptake regulator (Fur) controls the expression of iron-regulated genes via binding to a consensus sequence in the promoter regions in staphylococci [35]. Besides the *sbn* operon additional Fur-regulated genes and operons, as listed at the RegPrecise regulon site (http://regprecise.lbl.gov/RegPrecise/regulon.jsp?regulon_id=6608), exhibited slightly to moderately altered expression in SNM3 compared to BM. These included the *isd* genes (iron-regulated surface determinant system; also called *sir*; staphylococcal iron-regulated proteins) and those for the ferritin storage protein, the TatAC system and the ferrichrome ABC-transporter SstABCD (supplementary Figure S3).

**Figure 7:**
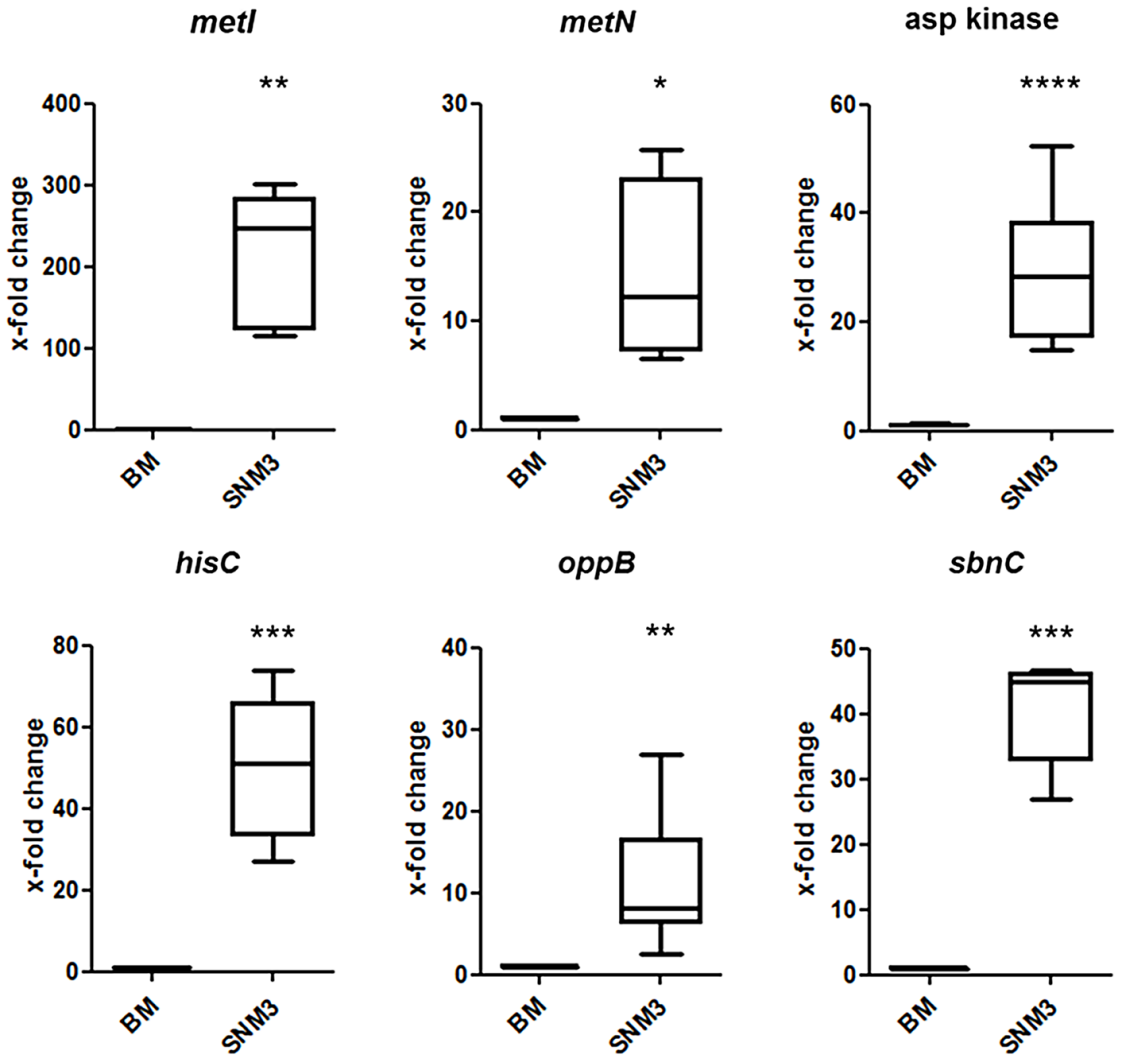
Validation of relative gene expression of selected genes up-regulated in the microarrays by qRT-PCR. Results from at least six independent BM or SNM3 cultures each are depicted as the x-fold change of expression of the respective genes in SNM3 vs. BM. Analyzed transcripts represent cysteine-γ-synthase (*metI*), a methionine transporter (*metN*), aspartate kinase, histidine biosynthesis gene (*hisC*), oligopeptide permease (*oppB*), and staphyloferrin B biosynthesis gene (*sbnC*). The upper and lower box limits and the horizontal lines within the boxes represent 25 and 75% percentiles and the means, respectively. The whiskers of the plots indicate minimum and maximum range. Significant differences calculated by the unpaired, two-tailed Student's *t* test with Welch's correction are indicated: * p≤0.05; **p≤0.01; *** p≤0.001; **** p≤0.0001.

To evaluate the validity of the microarray results, gene expression of selected *S. aureus* USA300 genes, which were strongly up-regulated in SNM3, was reinvestigated by qRT-PCR. Besides *metI* (methionine biosynthesis; SAUSA300_0360) and the methionine transporter gene *metN* (SAUSA300_0435), expression was analyzed for *hisC* (histidine biosynthesis; SAUSA300_2610), aspartate kinase (SAUSA300_1225), oligopeptide transporter *oppB* (SAUSA300_0201) and staphyloferrin B biosynthesis gene *sbnC* (SAUSA300_0120). As shown in Figure 7, all of the investigated genes exhibited significant up-regulation in SNM3 compared to BM, thereby confirming the microarray data.

### The methionine-biosynthetic pathway represents a potential target for antimicrobial strategies

The strong expression of methionine-biosynthetic genes prompted us to investigate if the absence of methionine is significantly limiting the growth of *S. aureus* USA300 in SNM3. However, the step-wise increase of methionine in SNM3 had no effect on colony formation on SNM3 agar (Fig. 3C) and only a marginal impact on *S. aureus* growth in liquid SNM3 (data not shown). Hence, methionine limitation does not compromise *S. aureus* growth, probably as a result of efficient ways to synthesize methionine. We also investigated if methionine limitation might be a reason for the inefficient outgrowth of CoNS on SNM3 agar. For this purpose, we analyzed colony formation of various nasal CoNS isolates and the laboratory strain *S. epidermidis* 1457 on SNM3 agar with increasing methionine concentrations (Fig. 3C). However, even high methionine amounts of 100 µM increased colony formation of *S. epidermidis* and CoNS strains only slightly, and only a small minority of strains benefitted strongly from the addition of methionine. This finding suggests that the inability of CoNS to thrive on SNM3 agar is probably the result of complex differences in the metabolic or regulatory properties of *S. aureus* and CoNS.

In order to analyze the importance of the capacity of *S. aureus* to synthesize methionine, the gene of the methionine-biosynthetic enzyme MetI was inactivated in *S. aureus* Newman. The resulting mutants showed no growth defects in complex medium. However, they were completely unable to grow in SNM3, while addition of methionine restored growth of the mutants, thereby demonstrating a crucial role of MetI under colonization-related conditions (Supplementary Fig. S4). Antimicrobial targets for new decolonization drugs are urgently needed because of increasingly emerging mupirocin resistant *S. aureus* [23]. The synthetic compound DL-propargylglycine has been shown to inhibit the bacterial cytathionine-γ-synthase MetI leading to a block of methionine biosynthesis [36]. We hypothesized that it may have antimicrobial activity against *S. aureus* under conditions where MetI plays a crucial role. DL-propargylglycine hardly affected growth of *S. aureus* USA300 in BM (minimal inhibitory concentration, MIC>10 mg/ml), whereas it inhibited growth of all tested *S. aureus* strains in SNM3 (Table 3). These data suggest that MetI inhibitors might in fact be useful to limit the growth of *S. aureus in vivo*. Supplementation of SNM3 with methionine abrogated the antimicrobial activity of DL-propargylglycine (MIC>10 mg/ml) confirming that DL-propargylglycine inhibits *S. aureus* growth by blocking an indispensable step of methionine biosynthesis.

**Table 3:** MIC values of DL-propargylglycine for *S. aureus* strains in complex medium (BM) and SNM3.

Strain	MIC in BM [mg/ml]	MIC in SNM3 [mg/ml]	MIC in SNM3 + 50 µM methionine [mg/ml]
*S. aureus* Newman	>10	0.09	>10
*S. aureus* USA300 LAC	>10	0.78	>10
*S. aureus* Dm*	>10	0.58	>10
*S. aureus* Kf*	>10	0.28	>10
*S. aureus* Mz*	>10	0.80	>10
*S. aureus* Sr*	>10	0.41	>10
*S. aureus* Cw*	>10	0.36	>10
*S. aureus* Bw*	>10	0.70	>10

, Nasal isolates from this study, for which *in vivo* gene expression was also determined.

### *metI* is strongly expressed during *S. aureus* colonization of the human nose and is crucial for efficient nasal colonization of cotton rats

**Figure 8:**
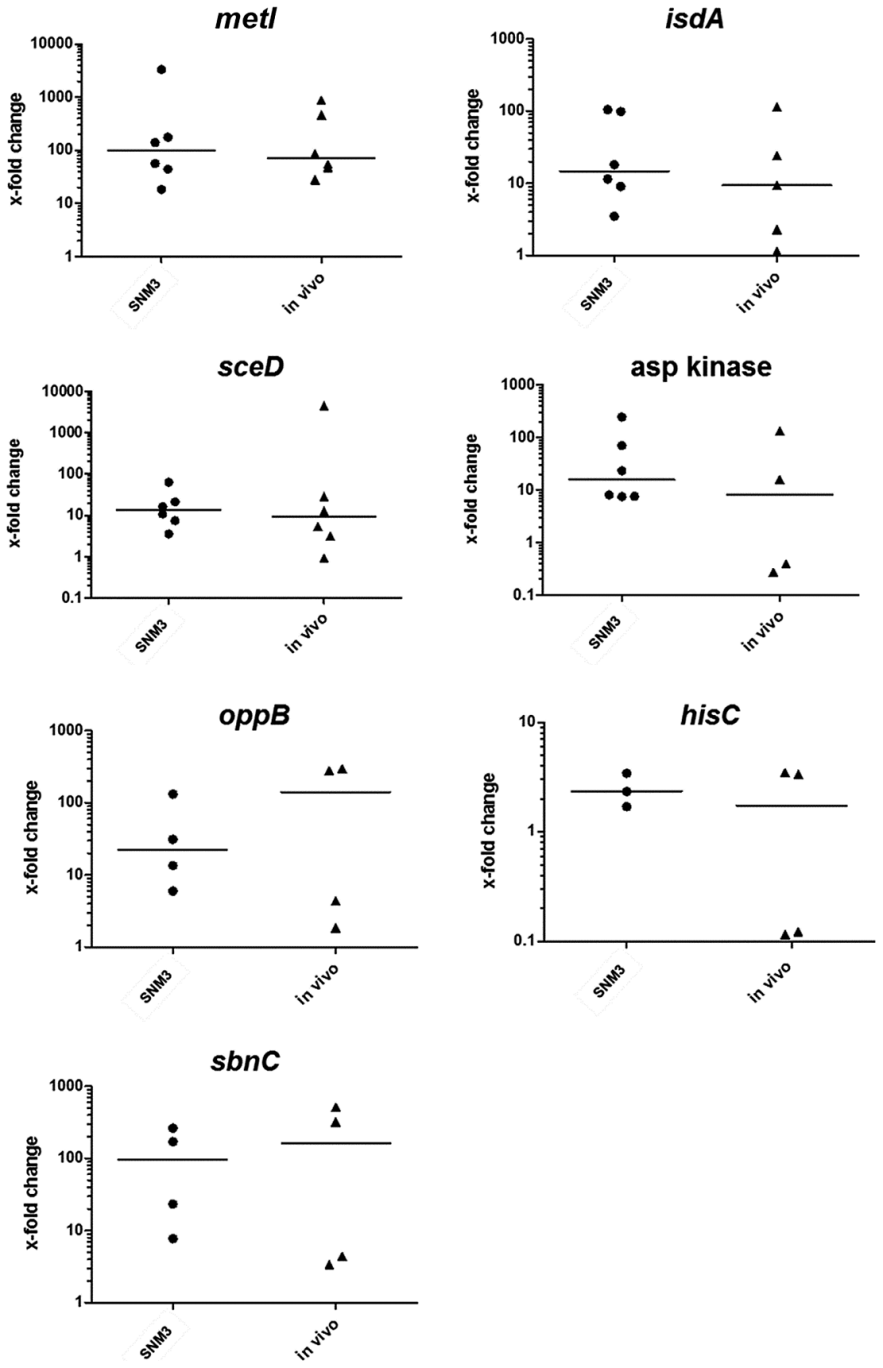
Relative expression of selected genes in nasal swabs from six *S. aureus* carriers (*in vivo*) compared to growth of the corresponding strains in BM or SNM3. The relative gene expression levels in nasal swabs (*in vivo*) and SNM3 cultures measured by qRT-PCR were compared with mRNA level of the corresponding strain in BM, which was defined as 1. Each symbol per group (SNM3 or *in vivo*) represents an independent nasal isolate. The means per group are indicated as horizontal lines. Less than six data points per group are presented for some of the genes since some samples yielded no expression signals. Analyzed transcripts represent cysteine-γ-synthase (*metI*), iron-regulated surface determinant A (*isdA*), a lytic transglycosylase (*sceD*), aspartate kinase, oligopeptide permease (*oppB*), histidine biosynthesis gene (*hisC*), and staphyloferrin B biosynthesis gene (*sbnC*).

In order to analyze if the critical genes found to be up-regulated by *S. aureus* USA300 in SNM3 vs. BM exhibit similar expression profiles during nasal colonization, their transcription was measured> by qRT-PCR in nasal swabs from six documented *S. aureus* carriers. Their corresponding nasal *S. aureus* strains were subsequently grown in SNM3 and BM for RNA isolation, and all samples were analyzed by qRT-PCR (Fig. 8). For some samples expression of certain genes was not detectable, possibly as a result of sequence variation at primer binding sites or low RNA concentrations.

The expression patterns in SNM3 and *in vivo* were overall very similar, and none of the analyzed genes exhibited significant differences *in vivo* and in SNM3 compared to BM. Gene expression in the six nasal isolates was generally more variable *in vivo* than in SNM3, suggesting that some parameters of the nasal living conditions may vary to a certain extent between donors. In most of the samples *isdA*, *sceD*, and *oppB* expression analysis confirmed up-regulation *in vivo* and in SNM3 compared to BM. While all strains that yielded detectable qRT-PCR signals for aspartate kinase and *hisC* were consistently up-regulated in SNM3 compared to BM, this was only found in some of the *in vivo* samples. This variability indicates that *in vivo* expression of these genes depends on the individual host. Expression of *sbnC* was high in SNM3 and *in vivo* compared to complex medium, confirming that iron-limiting conditions were indeed present under both conditions. Notably, expression of *metI* was strongly up-regulated in SNM3 compared to BM in all six strains (median about 100-fold, Fig. 8), and the *in vivo metI* expression was nearly the same as in SNM3.

**Figure 9:**
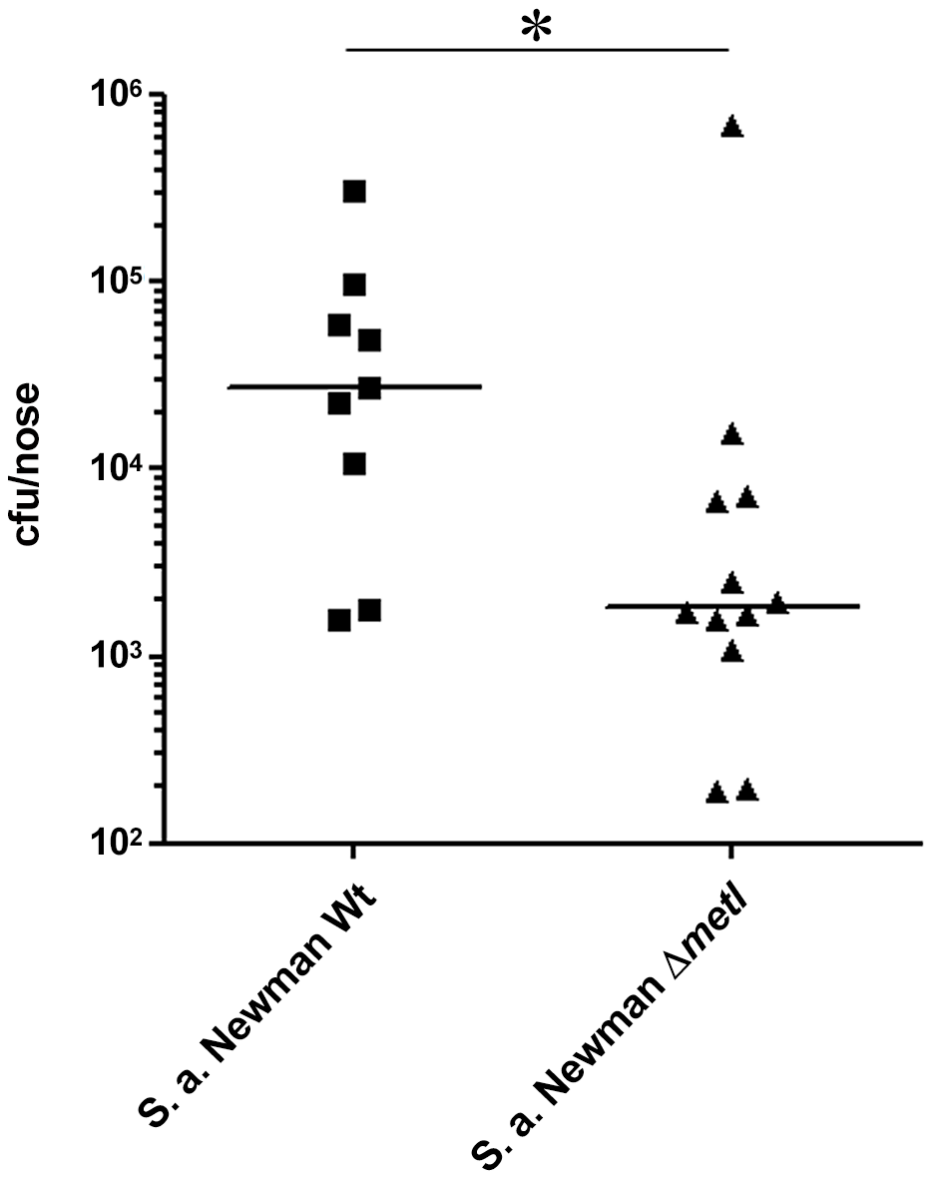
Cotton rat nasal colonization model. Bacterial numbers were determined six days after intranasal inoculation of cotton rats with 1×10^8^ colony forming units (CFU) of *S. aureus* (Newman). After six days the noses were dissected and bacterial CFUs were enumerated on *S. aureus*-selective HighChrome agar. Statistical analysis was performed by D'Agostino & Pearson omnibus normality test and a subsequent Mann Whitney test. Significant differences between groups are indicated by one (*P*<0.05) asterisk (*).

Cotton rats have been shown to be a suitable model for *S. aureus* nasal colonization [11], [37]. When S. *aureus* Newman and the isogenic Δ*metI* mutant were used to inoculate the noses of cotton rats, a strongly reduced colonization capacity of the mutant was observed compared to the parental strain (Figure 9). Thus, MetI has a critical role during nasal colonization, and the methionine-biosynthetic pathway may include previously unrecognized targets for new antimicrobial strategies.

## Discussion

The metabolomics analysis of nasal secretions reveals that the human nose represents an environment with rather limited nutrient availability. The concentrations of glucose and amino acids are substantially lower in nasal secretions compared to human plasma of healthy individuals (glucose about 0.04–1 mM vs. 4–8 mM; amino acids about 0.65–2.2 mM vs. 2.6 to 4.3 mM, respectively) [38]. The sputum covering lung epithelia of cystic fibrosis (CF) patients, which are frequently infected by *S. aureus* [39], [40], contains similar concentrations of glucose and even higher concentrations of free amino acids than plasma (1.3 to 4.5 mM and 4.7 to 24.7 mM, respectively [41]. These differences in nutrient availability suggest that *S. aureus* requires different metabolic activities during colonization of the human nose or infection of sterile tissues. It is interesting to note that lactate, an abundant compound on skin with concentrations around 2.5 mM [42] and a product of *S. aureus* energy metabolism [43], was undetectable in nasal secretions. Accordingly, metabolites in the nasal habitat differ from those on skin, and *S. aureus* metabolism does not seem to affect much the nasal metabolome. Our data indicate that the concentration of many nutrients in nasal secretions varies between donors, but none of the differences could be associated with the *S. aureus* carrier status. Thus, factors other than nutrient supply, such as differences in epithelial immunity or ligands for *S. aureus* adhesins, may be responsible for the predisposition to the *S. aureus* carriage status.

Various chemically defined media have been developed over the past decades with the purpose to achieve maximal growth and high capsule or protein expression in *S. aureus* [44]–[47]. In contrast, our aim was to simulate the *in vivo* situation in the human nose, to use a representative synthetic nasal medium for comparing colonization capacities of different staphylococcal strains and to elucidate colonization-related bacterial metabolic processes. Expression profiles of representative *S. aureus* genes upon growth in the artificial nasal medium SNM3 corresponded well to those found in the human nose indicating that SNM3 provides suitable conditions for simulating *S. aureus* growth during nasal colonization. Of note, global transcriptomes of bacteria from SNM3 and complex media differed extensively indicating that complex media can hardly reflect *in vivo*-related gene expression. In accord with this finding, transcriptome data of *S. aureus* grown in human blood and serum have also yielded major differences compared to previously described expression profiles from complex-media grown bacteria [48], [49]. Thus, SNM3 will allow to investigate bacterial metabolic processes during nasal colonization in detail and to monitor *S. aureus* competition with other resident bacteria under *in vivo*-like conditions. Nevertheless, results obtained with SNM3 in genome-wide *in vitro* experiments should be validated in *in vivo* models.

In accordance with the limited nasal nutrient supply, the human nasal microbiome has been shown to be much less complex [31], [50] than those of the upper or lower digestive tract, where bacteria are in regular contact with ingested food [30], [51]. Hence, bacteria in the human nose can be expected to compete fiercely for available nutrients and depend on mechanisms allowing them to thrive even with very low amounts of nutrients. In line with this notion all tested nasal *S. aureus* isolates grew well in SNM3, and almost all viable cells formed colonies on SNM3 agar. *S. aureus* is obviously adapted to growth in moist environments with very dilute nutrients, while human skin is usually dry except for atopic dermatitis patients, whose skin structure and permeability is severely perturbed [52]. In contrast, CoNS colonize healthy skin as their major habitat [53], which is in accordance with our finding that most of the tested CoNS isolates had major problems to form colonies on SNM3 agar and grew in liquid SNM3 only after long lag phases. It has remained unclear if CoNS use the human nose as a preferred or only as a transient habitat. While permanent *S. aureus* carriers are usually colonized by one specific single clone, multiple *S. epidermidis* strains can be found per nose and the pattern of strains is highly variable over time [54]. Our results indicate that CoNS are usually much less adapted to growth in nasal secretions than *S. aureus*. In line with our data a recent study has found a negative association between the colonisation with *S. aureus* and the abundance of *S. epidermidis*. It has been speculated that these bacterial species compete with each other during colonization of the nares [31]. Our data supports the notion that most *S. aureus* strains can overgrow CoNS because of better metabolic adaptation. In addition to differences in the abilities to utilize dilute nutrients, competition between *S. aureus* and CoNS may also involve different capacities to produce bacteriocins such as lantibiotics [55], [56], to induce and resist host antimicrobial peptides such as defensins [57], which have been detected in nasal secretions [58], and to produce factors that compromise the ability of other staphylococci to adhere to epithelial surfaces such as the *S. epidermidis* Esp protease [59].

Metabolomics analyses have been proposed to facilitate the identification of new antimicrobial targets and have recently helped to define the staphylococcal pyruvate dehydrogenase as a target for a new class of organobismuth antimicrobials [60]. We demonstrate here that a combined metabolomics and transcriptomics approach can lead to the identification of targets that are specifically important during *in vivo*-like conditions. Gene expression analysis of *S. aureus* grown in SNM3, or from *in vivo* samples, revealed that various amino acid biosynthesis operons are up-regulated during colonization. This implies a general importance of several anabolic pathways under such conditions.

Since SNM3 does not contain isoleucine, it can be assumed that the global transcription repressor CodY plays an important role for the up-regulation of a number of genes under the conditions used for microarray experiments, especially those for isoleucine biosynthesis [61], [62]. It has recently been shown that CodY also influences *S. aureus metICFE-mdh* expression, which is essentially regulated by a T-box riboswitch that recognizes uncharged initiator tRNA^fMet^ [63]. Despite the obvious influence of CodY on the gene expression pattern no clear signs of stringent response, like down-regulation of ribosomal protein *rpsL*, could be detected [64].

The absence of methionine in nasal secretions and SNM3 was reflected by the strong up-regulation of *S. aureus* methionine import and, most conspicuously, methionine biosynthesis genes in SNM3 and in the noses of human volunteers. In a similar approach, metabolomics analysis of sputum from CF patients has recently allowed to develop a synthetic sputum medium, leading to the unexpected finding that *Pseudomonas aeruginosa* depends on L-alanine as carbon source during CF lung infections. Furthermore, the high concentrations of aromatic amino acids in CF-sputum and the corresponding synthetic medium have been implicated in high-level production of pyocyanin [41], [65]. This cytotoxic respiratory inhibitor is important in competition of *P. aeruginosa* with *S. aureus* in the CF lung, since the latter is strongly inhibited because of its pyocyanin-sensitive cytochrome bd oxidase [66].

The methionine biosynthetic enzymes fulfil important criteria for antimicrobial drug targets, because they are absent from human cells that need to take up methionine from exogenous sources. However, such enzymes have hardly been regarded as antimicrobial targets before, because their importance has probably been missed when growing bacteria in complex media in antimicrobial screening programs. Nevertheless, this pathway has recently been proposed as a potential staphylococcal “Achilles heel” [63]. In our study the synthetic compound DL-propargylglycine, described as an inhibitor of cystathionine-γ-synthase (MetI), which is unique to microorganisms and plants [36], [67] was used. The activity of DL-propargylglycine against *S. aureus* and the inability of *metI* mutants to grow in SNM3 underscore the potential use of MetI or other methionine-biosynthetic enzymes as targets for new nasal decolonisation drugs. These drugs could be alternatives to eradicate e.g. mupirocin-resistant *S. aureus*. In agreement with this notion, the *S. aureus metI* gene was strongly upregulated in human noses, and the *metI* mutant was compromised in nasal colonization of cotton rats. However, DL-propargylglycin itself does not seem to be suitable as a drug, because it also inhibits the human cystathionine-γ-lyase. This enzyme converts exogenous methionine to cysteine via the transsulfuration pathway [68], [69], leading to reduced production of the gaseous messenger molecule hydrogen sulfide with various eminent consequences [70]. While the rather low activity against *S. aureus* and the severe impact on mammalian metabolism precluded the use of DL-propargylglycine in animal models, derivatives or new compounds with higher selectivity and activity against bacterial MetI or other methionine-biosynthetic enzymes may become promising lead substances for the development of new antimicrobial drugs. Notably, all these enzymes were significantly up-regulated in SNM3 (Fig. 6).

## Materials and Methods

### Ethics statement

The nasal secretion study and sample collection procedures were approved by the clinical ethics committee of the University of Tuebingen (No. 109/2009 BO2) and informed written consent was obtained from all volunteers. Secretion samples and nasal swabs were taken exclusively from healthy adults.

### Strains and growth conditions

The staphylococcal laboratory strains used in this study are *S. aureus* USA300 LAC [71], *S. aureus* Newman [72], and *S. epidermidis* 1457 [73]. Beside these characterised strains a set of 87 staphylococcal isolates from nasal swabs from 37 healthy volunteers were used. The collection of nasal isolates includes 18 *S. aureus*, 57 *S. epidermidis*, six *S. capitis*, three *S. lugdunensis*, two *S. warneri* and one *S. hominis* strain. Identification was accomplished according to an established scheme [74] by sequencing of a variable ca. 931-bp PCR fragment of the glyceraldehyde-3-phosphate dehydrogenase gene (*gap*), amplified with primer pair gap-F and gap-R (Table S1). Ambiguities were clarified by additional sequencing of a variable part of the *dnaJ* gene, amplified with primer pair dnaJ-F and dnaJ-R (Table S1).

BM (1% tryptone, 0.5% yeast extract, 0.5% NaCl, 0.1% glucose and 0.1% K_2_HPO_4_, pH 7.2) was used as standard complex medium. The composition of the chemically defined medium SNM, corresponding to nasal secretions, is listed in Tables 1 and 2. The content of inorganic ions in human nasal secretions listed in Table 1 has been published earlier [24], [25] and the described buffer, salt and co-factor concentrations were included in SNM as listed in Table 2. In contrast to the published data calcium was omitted from the medium, because it led to precipitates. Trace elements and cofactors were added from 1000-fold concentrated stock solutions. The iron-complexing agent 2, 2′-bipyridine (Merck, Darmstadt, Germany) was added at a final concentration of 200 µM. SNM3 contained the same concentrations of inorganic salts, urea, trace elements, and cofactors as SNM while amino acids, organic acids, and glucose as listed in Table 1 were threefold concentrated. BM and SNM agar plates contained 1.5% agar. For growth curves bacteria from overnight cultures grown in BM were centrifuged, washed with PBS, and diluted in SNM3 to an initial OD_600 nm_ of 0.02 in microtiter plates (MTP) and grown in a TECAN Infinite 200 PRO reader (Tecan Group Ltd., Switzerland) with shaking (180 rpm) at 37°C with continuous measurement of optical densities. For MIC determination 24-well MTP plates, essentially inoculated as described above, were grown for 48 hours at 37°C and 160 rpm. For the calculation of growth, inoculation density was subtracted from final optical density and the MICs, defined as the concentration of DL-propargylglycine (Sigma-Aldrich, Taufkirchen, Germany) at which 75% growth inhibition occurred, were calculated. For monitoring bacterial growth in three to 20-fold SNM cultures 12-ml medium in 100-ml buffled Erlenmeyer flasks were inoculated with an SNM3 over-night culture to an initial OD_578 nm_ of 0.02 and incubated with vigorous shaking at 160 rpm at 37°C, until the final OD_578 nm_ was determined after 90 h growth. For RNA isolation all media were inoculated with SNM3 overnight cultures, grown at 37°C with vigorous shaking at 160 rpm in 250-ml buffled Erlenmeyer flasks.

For continuous cultures sterile fresh medium was added from a reservoir to the growing culture with a peristaltic pump. A second pump was used to remove consumed medium, including bacteria, from the culture. 100 ml SNM in 500-ml glass bottles (Schott) were inoculated to an OD_578 nnm_ of 0.02 and incubated with shaking at 140 rpm at 37°C with a sterile filter in the bottle lid to allow aeration. About 4 ml fresh medium were added and simultaneously removed from the culture per hour resulting in exchange of the complete culture volume within approximately 24 hours. Non-continuous batch cultures were performed in the same way, except that continuous medium exchange was omitted.

### Collection of nasal secretions and metabolite analysis

Nasal secretions were taken with the help of slight vacuum suction with suction catheters (model 14 Ch, Bicakcilar Healthcare Products, Turkey) mounted on sputum collection traps (P. J. Dahlhausen & Co.GmbH, Germany). After short centrifugation the samples were immediately frozen at −80°C. Metabolites from frozen nasal secretions were extracted with methanol/chloroform/water 4/4/2.85 after addition of internal standards (ribitol and norvaline), samples were vortexed twice for 10 s and then centrifuged (4°C, 10 min, 13000 rpm). Supernatants were transferred to new glass vials and dried by lyophilisation. Dry samples were derivatized for GC-MS analyses and measured according to a previously described method [75]. Briefly, metabolites were identified by matching retention time and identification ion of pure chemical standards, measured under the same conditions. Ratios of peak areas to the respective internal standard (ribitol) were used for absolute and relative quantification. Calibration curves of pure substances were measured over a wide range of concentrations under the same conditions and were used for the calculation of the total, micromolar metabolite concentration.

Because arginine is unstable when derivatized for GC-MS, its content was determined by HPLC using ortho-phthaldehyde (OPA) pre-column derivatization. OPA was diluted to a final concentration of 1 mg/ml with 1 M sodium-borate-buffer, pH 9.0. Nasal secretion samples were sonicated for 20 s in a sonication water bath to reduce viscosity and diluted 1∶1 with distilled water. Subsequently, each sample (6 µl) was mixed with 1.5 µl OPA for 90 s and immediately injected and separated on an Agilent 1200 series HPLC-system using a Grom-SIL OPA-3 (5 µm), 4.0×150 mm column (Grace Davison, Lokeren, Belgium). A linear gradient from 100% buffer A (25 mM sodium-phosphate buffer, 0.7% tetrahydrofuran, pH 7.2) to 100% buffer B (50% buffer A, 35% methanol, 15% acetonitrile) in 24 min was run at a flow rate of 1.1 ml/min. Arginine was detected at 450 nm and quantified against a standard calibration curve with arginine concentrations of 10 µM, 100 µM, 1 mM, and 10 mM. Data were analyzed by Agilent ChemStation software.

Relative abundance is given in cases were no dilution series of pure standard compounds was measured. Data analysis was performed within the GC-MS software Chemstation (Vers. E.02.00 Service Pack 2, Agilent). Statistical analysis was accomplished with Aabel 3.0.4 (Gigawiz) and SIMCA P+ 12.0.1, for principal component analysis (PCA), and for partial least square (PLS) analysis log-transformed peak areas with UV scaling were used.

### RNA isolation and amplification

For qRT-PCR 40-ml cultures were inoculated to an optical density of 0.02 at 578 nm (ca. 2×10^7^ CFU/ml) and grown for three hours. RNA was isolated by a modified protocol of Bhagwat et al. adapted to large culture volumes [76]. Briefly, cells were immediately killed and RNA was stabilized by the addition of 1/9 vol. of an ice-cold 9∶1 ethanol∶phenol solution (equilibrated with Tris/EDTA-buffer (TE); Applichem, Darmstadt, Germany). After 5 min on ice cells were harvested (20 min, 4500×g, 4°C), resuspended in 1 ml TRIZOL solution (Invitrogen - Life Technologies Corporation, Darmstadt, Germany), and lysed with 0.5 ml zirconia-silica beads (Karl Roth GmbH, Karlsruhe, Germany; 0.1 mm-diameter) in a high-speed benchtop homogenizer (FastPrep-24, MP Biomedicals, Germany). Subsequently, RNA was isolated as described in the instructions provided by the manufacturer of the RNA isolation kit (ExpressArt RNAready, AmpTec GmbH, Germany). In order to get rid of potential RT-PCR inhibitors, a first washing step with 0.5 ml ‘Inhibitor removal buffer’ was applied (High Pure PCR Template Preparation Kit, Roche Applied Science). DNA was removed by on-column DNAse treatment according to the ExpressArt RNAready protocol.

RNA for microarrays was isolated from 100-ml cultures, inoculated to an OD_578 nm_ of 0.005 and grown until OD_578 nm_ of 0.02. RNA was stabilized as described above by ethanol∶phenol addition. After centrifugation, the cell pellet was washed with 2 ml ice-cold ethanol∶acetone (1∶1) to remove phenol traces. A second washing and stabilisation step was applied by resuspending the cells in 2 ml RNAprotect bacteria reagent (QIAGEN GmbH, Germany)∶TE-buffer (2∶1). After centrifugation cells were lysed in 100 µl TE buffer containing 10 µl lysostaphin (2 mg/ml, Genmedics GmbH, Germany) by incubation for 3 min at room temperature. After the addition of 350 µl RLT buffer (RNeasy Kit, QIAGEN GmbH, Germany) cells were lysed with 0.2 ml zirconia-silica beads as described above and RNA was purified with the RNeasy Kit according to the manufacturer's instructions.

RNA for qRT-PCR from nasal swabs was isolated essentially as described above for *in vitro* cultures. The cotton swabs (MSP Schmeiser, Horb, Germany) were soaked in sterile PBS and used to carefully wipe the anterior nares of volunteers and afterwards directly resuspended in 1 ml TRIZOL. For confirming the carrier status additional swabs were taken, resuspended in PBS, and serial dilutions were plated on BM agar. The presence of *S. aureus* was confirmed with the Slidex Staph Plus latex agglutination test (bioMérieux Deutschland GmbH, Nürtingen, Germany) according to the manufacturer's instructions.

Since some of the volunteers had quite low numbers of *S. aureus* in their nose (<1.000 *S. aureus*/swab), the isolated RNA amounts were sometimes not sufficient for qRT-PCR. In such cases RNA was amplified once with the ‘Bacterial Nano mRNA amplification Kit’ (AmpTec GmbH, Hamburg, Germany) as described by the manufacturer.

### Reverse transcription and quantitative real-time PCR (qRT-PCR)

Relative quantification of various transcripts was performed as described previously [18]. Briefly, isolated RNA from cultures and nasal swabs was transcribed into complementary DNA using SuperScriptIII Reverse Transcriptase (Invitrogen) and 200 ng of random hexamer primers (Fermentas, St. Leon-Rot, Germany). For relative quantification standards were generated by PCR with the primers listed in Table S1 with *S. aureus* USA300 genomic DNA as template. All agarose gel-purified PCR products were used in 10-fold serial dilutions. For the genes *metN* and *sbnC* PauI (Fermentas) -digested and purified chromosomal DNA of *S. aureus* USA300 was used as standard. Complementary DNA was diluted 1∶10 and quantitative real-time PCR (qRT-PCR) was performed with primers listed in Supplementary Table S1, using the LightCycler instrument (LightCycler 480, Roche) in combination with SYBR Green I (QuantiFast SYBR Green PCR Kit, QIAGEN). Master mixes were prepared according to the manufacturer's instructions. Relative gene expression level was calculated by the method of Pfaffl with PCR efficiency correction [77].

### Microarray analysis

5 µg of RNA from three biological replicates per condition were applied to GeneChip microarrays (Affymetrix) and processed according to the manufacturer's protocol. The biological replicates yielded highly reproducible expression profiles.

The GeneChip *S. aureus* genome array was provided by MFTServices (www.mftservices.de), a Core Lab provider authorized by Affymetrix Inc. (Santa Clara, CA). GeneChip hybridization, washing, staining, and scanning were performed as described by the manufacturer. The images were processed with Expression Console (Affymetrix). The raw data from the array scans were normalized by median-centering genes for each array, followed by log transformation. Expressed genes were identified using Affymetrix GeneChip Operating Software (GCOS, Ver.1.1). To identify genes that are differentially expressed in treated samples compared to controls, the Partek software version 6.6 was used. To select the differentially expressed genes, we used threshold values of ≥2.0- and ≤−2.0-fold change between the conditions. The false discovery rate (FDR) significance level with Benjamini-Hochberg was <5%. The data discussed in this publication have been deposited in NCBI's Gene Expression Omnibus [78] and are accessible through GEO Series accession number GSE43712 (http://www.ncbi.nlm.nih.gov/geo/query/acc.cgi?acc=GSE43712).

### Construction of a cystathionine-γ-synthase mutant and colonisation of cotton rat nares

For the construction of a markerless mutant of *S. aureus* Newman, the flanking regions of *metI* were amplified with primer pairs cgsF1 up/cgsF1 down or cgsF2 up/cgsF2 down. After digestion of PCR product F1 with EcoRI/BglII and of PCR product F2 with BglII/NheI the two fragments were ligated into the previously described vector pBASE6 [64], digested with EcoRI and NheI and used to transform *E. coli* DC10B [79]. The resulting plasmid pBASE6-ΔmetI was isolated and directly transferred to *S. aureus* Newman, where the homologous recombination process resulted in *metI* mutants, which were confirmed by PCR analysis.

For the colonisation of cotton rats spontaneous streptomycin-resistant mutants of *S. aureus* Newman wild type and Δ*metI* were selected on BM agar plates with 500 µg/ml streptomycin. The cotton rat model was used as described earlier [11]. Cotton rats were anesthetized and instilled intranasally with 10 µl of 1×10^8^ colony-forming units (CFU) of *S. aureus*. Six days after bacterial instillation the animals were euthanized and noses were removed surgically. The noses were vortexed in 1 ml of PBS containing 0.5% Tween for 30 s. Samples were plated on appropriate agar plates (B-medium, sheep blood containing 250 µg/ml streptomycin and HiCrome Aureus Agar (Fluka)) and the bacterial CFU was determined. All animals received drinking water with 2.5 mg/ml streptomycin continuously, starting three days prior to the experiment to reduce the natural nasal flora. All animal experiments were conducted in accordance with German laws after approval (protocol T1/10) by the local authorities (Regierungspraesidium Tuebingen).

## Supporting Information

Figure S1 **Influence of continuous flow culture conditions on maximum cell numbers in synthetic nasal medium.** For continuous flow conditions sterile medium was added to the culture with a peristaltic pump (one culture volume within 24 hours), and the same volume was removed from the growing culture with a second peristaltic pump. Depicted are the highest CFU/ml values reached during cultivation (median CFU/ml with SEM of three independent cultures, each).
(DOCX)

Figure S2 **Heat maps of gene expression ratios of metabolism-related genes.** The COG functional classes of “Amino acid transport and metabolism” [E], “cell wall/membrane/envelope biogenesis” [M], and “Inorganic ion transport and metabolism” [P] are shown. The colors indicate the strength of up-regulation or down-regulation of genes in SNM3 compared to BM. All transcripts met the criteria for differentially regulated genes as described in the Materials and Methods section. Genes are quoted as loci of *S. aureus* USA300 and with their common names, where possible.
(DOCX)

Figure S3 **Gene expression ratios of iron (Fur)-regulated genes of** ***S. aureus*** **USA300 in SNM3.** To mimic the limited iron availability in the human nose, SNM3 was complexed with 200 µM 2, 2′-bipyridine. The table lists the expression levels of known Fur-regulated genes in SNM3 compared to complex medium (BM). The list of regulated genes was generated on the basis of the RegPrecise regulon information. (http://regprecise.lbl.gov/RegPrecise/regulon.jsp?regulon_id=6608).
(DOCX)

Figure S4 **Growth characteristics of** ***S. aureus*** **Newman and its isogenic** ***metI*** **mutant in complex medium and SNM3.** Representative growth curves for *S. aureus* Newman wild-type and two independently obtained *metI* mutants (1.1; 3.1) in complex B medium (A) or SNM3 (B). Addition of 50 µM methionine to SNM3 restored full growth of the mutants in SNM3.
(DOCX)

Table S1 **Oligonucleotides used in this study.**
(DOCX)
