# Supplementary material for: Nutrient Limitation Governs Staphylococcus aureus Metabolism and Niche Adaptation in the Human Nose
Source: PLoS Pathog. 2014 Jan 16;10(1):e1003862. doi: 10.1371/journal.ppat.1003862 (PMC3894218; doi:10.1371/journal.ppat.1003862)
Supplement: Table S1 — Oligonucleotides used in this study. (DOCX) [file ppat.1003862.s005.docx]

**Supplementary Table S1: Oligonucleotide primers**

| **Target gene** | **USA300_**  **annotation** | **Primer** | **Primer sequence 5’-3’** | **Purpose** |
| --- | --- | --- | --- | --- |
| *gap*  *dnaJ*  *gyrB* | SAUSA300_0756  SAUSA300_1539  SAUSA300_0005 | gap-F  gap-R  dnaJ-F  dnaJ-R  gyrB down  gyrB up  gyr297F  gyr574R | ATGGTTTTGGTAGAATTGGTCGTTTA  GACATTTCGTTATCATACCAAGCTG  GCCAAAAGAGACTATTATGA  ATTG(CT)TTACC(CT)GTTTGTGTACC  CGCTAAATTGTATCGTATTAATCAAGG  ctccttcaaaagttcagttcacagcgc  TTAGTGTGGGAAATTGTCGATAAT  AGTCTTGTGACAATGCGTTTACA | Species identification  Species identification  Species identification  Species identification  Standard construction Standard construction  qRT-PCR  qRT-PCR |
| *isdA*  RNAIII  cystathio-nine-γ-synthase  aspartate kinase  *psmβ*  *clfB*  *sceD*  *hisC*  *oppB*  *metN*  *sbnC* | SAUSA300_1029  Upstream of  SAUSA300_1989  SAUSA300_0360  SAUSA300_1225  SAUSA300_1068  SAUSA300_2565  SAUSA300_2051  SAUSA300_2610  SAUSA300_0201  SAUSA300_0435  SAUSA300_0120  Flanking region of  SAUSA300_0360  Flanking region of  SAUSA300_0360 | isdA down  isdA up  isdA-2665  isdA-2782  agr RNAIII down  agr RNAIII up  agr-1189  agr-1436  cys gamma syn down  cys gamma syn up  cys syn 277  cys syn 377  aspartate kinase down  aspartate kinase up  asp kin 318  asp kin 560  psm beta down  psm beta up  psmB268  psmB374  clfB down  clfB up  clfB-136  clfB-270  sceD down  sceD up  sceD-1404  sceD-1497  his bio down  his bio up  his bio 303  his bio 414  oppB down  oppB up  oppB582  oppB684  ABC 76  ABC 234  sbnC 221  sbnC 454  cgs-F1down  cgs-F1 up  cgs-F2down  cgs-F2 up | tattgttatacgaaaatagatgtgctag  CTACAGATGCAACGCCTAGTGATGAC  GCAGTTACAGTAGGTTTA  CAGCAAAACCAAACAATG  GAGCATGAATTTTTAACCGCTGGCG  gggcttctttttagttgctgcagg  CGATGTTGTTTACGATAGC  CGACACAGTGAACAAATTC  CATTGACTAATTAGCCTCCTTCG  ttacctcccgcttgttcaatatctg  GGTGCCAATGTAGAAATG  GCATCGTCAATAGTGGCT  ccaatgctactcaaattaaaaaggtt  gtaggtgagcaacgtattaaggatg  TTGAACGCATAACGATACTG  AAAATACGAACCGCCCTC  ATTTCGGTGATTTAAAAGCTACGC  ttttatatcccaatttccccatc  CAAAGGTGAGGGAGAGAT  CTACGATACTTGTGCCTAAT  CCACATCAGTAATAGTAGGGGCAAC  GTTCTTACCTGTATTGTCGTAATGCCC  ATAGGCAATCATCAAGCA  TGTATCATTAGCCGTTGTAT  GCATCATCATTAGCAGTAGGTTTAGG  GTGTTATATAATTTTACTGCTGCGG  GTGGTGCTTCAACTTCTT  CAACAGGTGCTAATGGAG  GGAAGCTTATGCTAAGTTTTACGG  GATAAATCATATTTTGCGCTCCATTC  ATTGTCTTGTCTCTTCTCTATG  ACTGCTGGAACGATAAAG  CATGATGTTGTGCCTCCCTATTCAC  CTAACGTTACAATTGGAATAATGGC  TGTCATCATTGGTGTTATTG  TATCTGTGCCTTTGTTGC  TTTACGGTCAATCGCAAT  CCTCATCATTTTATCGCTAT  TTACGATAGAGGGAAGGGT  ATGGATGAAATGGACGAT  TTTCGAATTCTTCAATCGCATTTAAGTC  gtgattagatctaactgtgtatccttcata  acgagatctcatccaagcattagataaagc  gtagtcgaccgtgagacattgttgctgga | Standard construction  Standard construction  qRT-PCR  qRT-PCR  Standard construction  Standard construction  qRT-PCR  qRT-PCR  Standard construction  Standard construction  qRT-PCR  qRT-PCR  Standard construction  Standard construction  qRT-PCR  qRT-PCR  Standard construction  Standard construction  qRT-PCR    qRT-PCR  Standard construction  Standard construction  qRT-PCR    qRT-PCR  Standard construction  Standard construction  qRT-PCR  qRT-PCR  Standard construction  Standard construction  qRT-PCR  qRT-PCR  Standard construction  Standard construction  qRT-PCR  qRT-PCR  qRT-PCR  qRT-PCR  qRT-PCR  qRT-PCR  knock-out construction  knock-out construction  knock-out construction  knock-out construction |
|  |  |  |  |  |
