# Supplementary material for: Nutrient Limitation Governs Staphylococcus aureus Metabolism and Niche Adaptation in the Human Nose
Source: PLoS Pathog. 2014 Jan 16;10(1):e1003862. doi: 10.1371/journal.ppat.1003862 (PMC3894218; doi:10.1371/journal.ppat.1003862)
Supplement: Supplementary file 2 [file ppat.1003862.s001.docx]

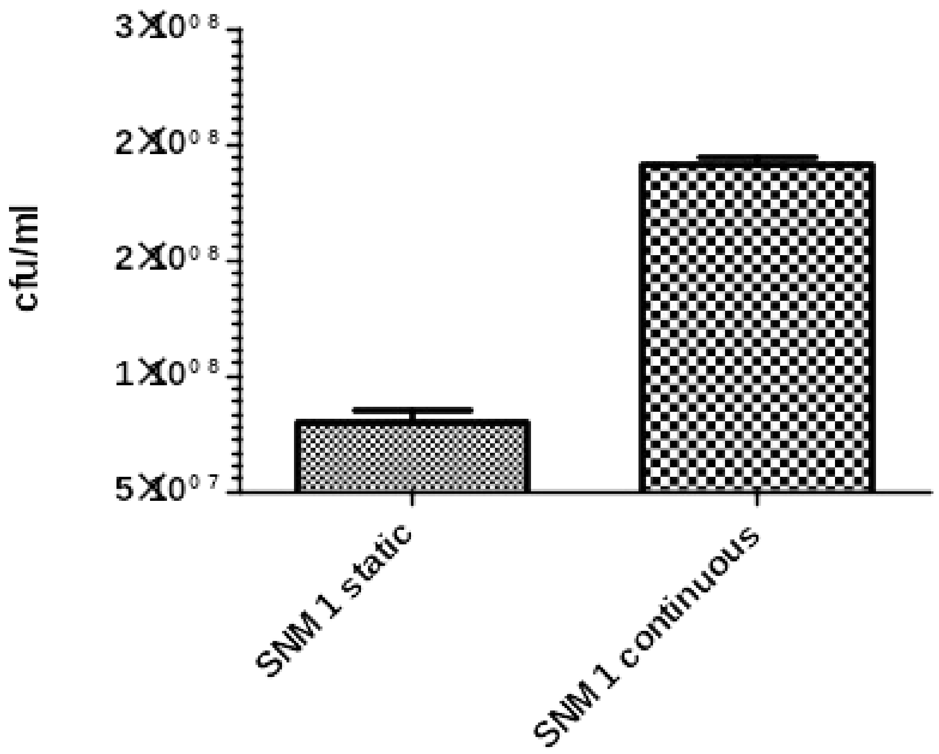

cfu/ml
3X10⁰ 8
2X10⁰ 8
2X10⁰ 8
1X10⁰ 8
5X10⁰ 7
SNM 1 static
SNM 1 continuous
