# Supplementary material for: Nutrient Limitation Governs Staphylococcus aureus Metabolism and Niche Adaptation in the Human Nose
Source: PLoS Pathog. 2014 Jan 16;10(1):e1003862. doi: 10.1371/journal.ppat.1003862 (PMC3894218; doi:10.1371/journal.ppat.1003862)
Supplement: Supplementary file 3 [file ppat.1003862.s002.docx]

			Regulated genes SNM vs. BM		
LOCUS USA300	Gene	Fold change	Function	Category	COG no.
SAUSA300_0010			Predicted branched-chain amino acid permease (azaleucine resistance)	[E]	COG1296
SAUSA300_0012	metX		Homoserine acetyltransferase	[E]	COG2021
SAUSA300_0118	sbnA		Cysteine synthase (staphyloferrin B biosynthesis)	[E]	COG0031
SAUSA300_0119	sbnB		Predicted ornithine cyclodeaminase (staphyloferrin B biosynthesis)	[E]	COG2423
SAUSA300_0125	sbnH		Diaminopimelate decarboxylase (staphyloferrin B biosynthesis)	[E]	COG0019
SAUSA300_0201	oppB2		ABC-type dipeptide/oligopeptide/nickel transport systems, permease components	[EP]	COG0601
SAUSA300_0202			ABC-type dipeptide/oligopeptide/nickel transport systems, permease components	[EP]	COG1173
SAUSA300_0203			ABC-type dipeptide transport system, periplasmic component	[E]	COG0747
SAUSA300_0204	ggt		Gamma-glutamyltransferase	[E]	COG0405
SAUSA300_0338			Lactoylglutathione lyase and related lyases	[E]	COG0346
SAUSA300_0357	metE		Methionine synthase II (cobalamin-independent)	[E]	COG0620
SAUSA300_0358	metF		Methionine synthase I (cobalamin-dependent), methyltransferase domain	[E]	COG0646
SAUSA300_0359	metC		Cystathionine beta-lyase	[E]	COG0626
SAUSA300_0360	metI		cystathionine gamma-synthase	[E]	COG0626
SAUSA300_0435	metN		D-methionine transport system ATP-binding protein	[E]	COG1135
SAUSA300_0436	metP		D-methionine transport system permease protein	[E]	COG2011
SAUSA300_0437	metQ		D-methionine transport system substrate-binding protein	[P]	COG1464
SAUSA300_0445	gltB		Glutamate synthase domain 1	[E]	COG0067
SAUSA300_0446	gltD		NADPH-dependent glutamate synthase beta chain and related oxidoreductases	[ER]	COG0493
SAUSA300_0708			Histidinol-phosphate/aromatic aminotransferase and cobyric acid decarboxylase	[E]	COG0079
SAUSA300_0796	metN2		D-methionine transport system ATP-binding protein	[E]	COG1135
SAUSA300_0797	metP2		D-methionine transport system permease protein	[E]	COG2011
SAUSA300_0798	metIQ2		D-methionine transport system substrate-binding protein	[E]	COG1464
SAUSA300_0860	rocD		Ornithine/acetylornithine aminotransferase	[E]	COG4992
SAUSA300_0887	oppB		ABC-type dipeptide/oligopeptide/nickel transport systems, permease components	[EP]	COG0601
SAUSA300_0888	oppC		ABC-type dipeptide/oligopeptide/nickel transport systems, permease components	[EP]	COG1173
SAUSA300_0889	oppD		ABC-type dipeptide/oligopeptide/nickel transport system, ATPase component	[EP]	COG0444
SAUSA300_0890	oppF		ABC-type oligopeptide transport system, ATPase component	[E]	COG4608
SAUSA300_0891	oppA		ABC-type oligopeptide transport system, periplasmic component	[E]	COG4166
SAUSA300_0892	oppA2		ABC-type dipeptide transport system, periplasmic component	[E]	COG0747
SAUSA300_0893	oppF2		ABC-type dipeptide/oligopeptide/nickel transport system, ATPase component	[EP]	COG0444
SAUSA300_0896	oppC2		ABC-type dipeptide/oligopeptide/nickel transport systems, permease components	[EP]	COG1173
SAUSA300_0914			Na+/alanine symporter	[E]	COG1115
SAUSA300_1063			Carbamate kinase	[E]	COG0549
SAUSA300_1225			Aspartokinases	[E]	COG0527
SAUSA300_1226	dhoM		Homoserine dehydrogenase	[E]	COG0460
SAUSA300_1227	thrC		Threonine synthase	[E]	COG0498
SAUSA300_1228	thrB		Homoserine kinase	[E]	COG0083
SAUSA300_1265	trpC		Indole-3-glycerol phosphate synthase	[E]	COG0134
SAUSA300_1266	trpF		Phosphoribosylanthranilate isomerase	[E]	COG0135
SAUSA300_1267	trpB		Tryptophan synthase beta chain	[E]	COG0133
SAUSA300_1268	trpA		Tryptophan synthase alpha chain	[E]	COG0159
SAUSA300_1286	lysC		Aspartokinases	[E]	COG0527
SAUSA300_1287	asd		Aspartate-semialdehyde dehydrogenase	[E]	COG0136
SAUSA300_1288	dapA		Dihydrodipicolinate synthase/N-acetylneuraminate lyase	[EM]	COG0329
SAUSA300_1289	dapB		Dihydrodipicolinate reductase	[E]	COG0289
SAUSA300_1290	dapD		Tetrahydrodipicolinate N-succinyltransferase	[E]	COG2171
SAUSA300_1293	lysA		Diaminopimelate decarboxylase	[E]	COG0019
SAUSA300_1300	braB		Branched-chain amino acid permeases	[E]	COG1114
SAUSA300_1331	ald		Alanine dehydrogenase	[E]	COG0686
SAUSA300_1565			Allophanate hydrolase subunit 2	[E]	COG1984
SAUSA300_1566			Allophanate hydrolase subunit 1	[E]	COG2049
SAUSA300_1662	nifZ		Cysteine sulfinate desulfinase/cysteine desulfurase and related enzymes	[E]	COG1104
SAUSA300_1669			Serine-pyruvate aminotransferase/archaeal aspartate aminotransferase	[E]	COG0075
SAUSA300_1683			Chorismate mutase	[E]	COG1605
SAUSA300_1976			Acetylornithine deacetylase/Succinyl-diaminopimelate desuccinylase and related deacylases	[E]	COG0624
SAUSA300_2006	ilvD		Dihydroxyacid dehydratase/phosphogluconate dehydratase	[EG]	COG0129
SAUSA300_2007	ilvB		acetolactate synthase , large subunit	[EH]	COG0028
SAUSA300_2008	ilvN		Acetolactate synthase, small (regulatory) subunit	[E]	COG0440
SAUSA300_2009	ilvC		Ketol-acid reductoisomerase	[EH]	COG0059
SAUSA300_2010	leuA		Isopropylmalate/homocitrate/citramalate synthases	[E]	COG0119
SAUSA300_2012	leuC		3-isopropylmalate isomerase large subunit	[E]	COG0065
SAUSA300_2013	leuD		3-isopropylmalate isomerase small subunit	[E]	COG0066
SAUSA300_2014	ilvA		Threonine dehydratase	[E]	COG1171
SAUSA300_2137			Predicted amino acid racemase	[E]	COG3457
SAUSA300_2291	gltS		Na+/glutamate symporter	[E]	COG0786
SAUSA300_2390	opuCD		ABC-type proline/glycine betaine transport systems, permease component	[E]	COG1174
SAUSA300_2392	opuCB		ABC-type proline/glycine betaine transport systems, permease component	[E]	COG1174
SAUSA300_2393	opuCA		ABC-type proline/glycine betaine transport systems, ATPase components	[E]	COG1125
SAUSA300_2414			Diaminopimelate epimerase	[E]	COG0253
SAUSA300_2497			Aspartate/tyrosine/aromatic aminotransferase	[E]	COG0436
SAUSA300_2538			Amino acid transporters	[E]	COG0531
SAUSA300_2539			4-aminobutyrate aminotransferase and related aminotransferases	[E]	COG0160
SAUSA300_2572	aur		Zinc metalloprotease (elastase)	[E]	COG3227
SAUSA300_2605	hisI		Phosphoribosyl-AMP cyclohydrolase	[E]	COG0139
SAUSA300_2606	hisF		Imidazoleglycerol-phosphate synthase	[E]	COG0107
SAUSA300_2607	hisA		Phosphoribosylformimino-5-aminoimidazole carboxamide ribonucleotide (ProFAR) isomerase	[E]	COG0106
SAUSA300_2608	hisH		Glutamine amidotransferase	[E]	COG0118
SAUSA300_2609	hisB		Imidazoleglycerol-phosphate dehydratase	[E]	COG0131
SAUSA300_2610	hisC		Histidinol-phosphate/aromatic aminotransferase and cobyric acid decarboxylase	[E]	COG0079
SAUSA300_2611	hisD		Histidinol dehydrogenase	[E]	COG0141
SAUSA300_2612	hisG		ATP phosphoribosyltransferase	[E]	COG0040

			Regulated genes SNM vs. BM		
LOCUS USA300	Gene	Fold change	Function	Category	COG no.
SAUSA300_0132			Glycosyltransferase	[M]	COG0438
SAUSA300_0152	capA		capsular polysaccharide synthesis enzyme Cap5A	[M]	COG3944
SAUSA300_0155	capD		capsular polysaccharide synthesis enzyme Cap5D	[MG]	COG1086
SAUSA300_0156	capE		capsular polysaccharide synthesis enzyme Cap8E	[MG]	COG1086
SAUSA300_0157	capF		capsular polysaccharide synthesis enzyme Cap5F	[MG]	COG0451
SAUSA300_0158	capG		capsular polysaccharide synthesis enzyme Cap5G	[M]	COG0381
SAUSA300_0269			Penicillin V acylase and related amidases	[M]	COG3049
SAUSA300_0651			CHAP domain containing secretory antigen ssaA homolog	[M]	COG1388
SAUSA300_1030	isdC		heme uptake protein	[M]	COG5386
SAUSA300_1089	lsp		Lipoprotein signal peptidase	[MU]	COG0597
SAUSA300_1292			Alanine racemase	[M]	COG0787
SAUSA300_2145	opuD		Choline-glycine betaine transporter opuD homolog	[M]	COG1292
SAUSA300_2391	opuCC		glycine betaine/choline-binding (lipo)protein of an ABC-type transport system	[M]	COG1732
SAUSA300_2500	crtQ		hypothetical glycosyltransferase involved in cell wall biogenesis	[M]	COG0463

			Regulated genes SNM vs. BM		
LOCUS USA300	Gene	Fold change	Function	Category	COG no.
SAUSA300_0174			ABC-type nitrate/sulfonate/bicarbonate transport system, ATPase component	[P]	COG1116
SAUSA300_0175			ABC-type nitrate/sulfonate/bicarbonate transport systems, lipoprotein	[P]	COG0715
SAUSA300_0176			ABC-type nitrate/sulfonate/bicarbonate transport system, permease component	[P]	COG0600
SAUSA300_0344	efeO		iron uptake system component EfeO, putative lipoprotein	[P]	COG2822
SAUSA300_0345	efeB		deferrochelatase/peroxidase EfeB, tat-translocated protein	[P]	COG2837
SAUSA300_1032	SirF		ABC-type Fe^{3+}-hydroxamate transport system, periplasmic component	[P]	COG0614
SAUSA300_1033	SirG/isdF		ABC-type Fe^{3+}-siderophore transport system, permease component	[P]	COG0609
SAUSA300_1561	mntH		Mn^{2+} and Fe^{2+} transporters of the NRAMP family	[P]	COG1914
SAUSA300_1874	ferritin		Ferritin-like protein	[P]	COG1528
SAUSA300_1996	amt		Ammonia permease	[P]	COG0004
SAUSA300_2034	kdpA		K^+-transporting ATPase, A chain	[P]	COG2060
SAUSA300_2616			ABC-type cobalt transport system, permease component CbiQ and related transporters	[P]	COG0619
SAUSA300_2617			ABC-type cobalt transport system, ATPase component	[P]	COG1122

	>20	
	10-20	
	5-10	
	3-5	
increase	2-3	
decrease	2-3	
	3-5	
	5-10	
	10-20	
	>20	
