# Supplementary material for: Nutrient Limitation Governs Staphylococcus aureus Metabolism and Niche Adaptation in the Human Nose
Source: PLoS Pathog. 2014 Jan 16;10(1):e1003862. doi: 10.1371/journal.ppat.1003862 (PMC3894218; doi:10.1371/journal.ppat.1003862)
Supplement: Supplementary file 4 [file ppat.1003862.s003.docx]

Mu50 LOCUS	USA300 LOCUS	gene	function	x-fold SNM
SAV0113	SAUSA300_0115	sirC	iron complex transport system permease protein	1,17080705
SAV0114	SAUSA300_0116	sirB	iron complex transport system permease protein	1,23718107
SAV0115	SAUSA300_0117	sirA	iron complex transport system substrate-binding lipoprotein	1,3304276
SAV0116	SAUSA300_0118	sbnA	probable cysteine synthase A protein	11,8033407
SAV0117	SAUSA300_0119	sbnB	probable ornithine cyclodeaminase protein	9,69143299
SAV0118	SAUSA300_0120	sbnC	hypothetical protein	11,1778695
SAV0119	SAUSA300_0121	sbnD	hypothetical protein	5,59066539
SAV0120	SAUSA300_0122	sbnE	hypothetical protein	10,6733661
SAV0121	SAUSA300_0123	sbnF	similar to siderophore biosynthesis protein	6,79661624
SAV0122	SAUSA300_0124	sbnG	hypothetical protein	3,13793036
SAV0123	SAUSA300_0125	sbnH	probable diaminopimelate decarboxylase protein	6,98771128
SAV0124	SAUSA300_0126	sbnI	hypothetical protein	5,51628374
SAV0343	SAUSA300_0344	efeO	iron uptake system component EfeO (lipoprotein)	2,52614363
SAV0344	SAUSA300_0345	efeB	deferrochelatase/peroxidase EfeB (Tat system secreted)	2,04150061
SAV0345	SAUSA300_0346	efeU	high affinity iron permease EfeU	1,89739932
SAV0346	SAUSA300_0347	tatC	sec-independent protein translocase protein TatC	0,63320008
SAV0347	SAUSA300_0348	tatA	sec-independent protein translocase protein TatA	0,64119126
SAV0647	SAUSA300_0633	fhuA	ferrichrome transport ATP-binding protein	1,28416151
SAV0648	SAUSA300_0634	fhuB	ferrichrome transport permease	1,24212182
SAV0649	SAUSA300_0635	fhuG	ferrichrome transport permease	1,10604749
SAV0733	SAUSA300_0718	sstA	ferrichrome ABC transporter permease	1,02046498
SAV0734	SAUSA300_0719	sstB	ferrichrome ABC transporter permease	0,93055285
SAV0735	SAUSA300_0720	sstC	ferrichrome ABC transporter ATP-binding protein	0,77067794
SAV0736	SAUSA300_0721	sstD	iron transport substrate binding lipoprotein	0,87211482
SAV0830	SAUSA300_0788		hypothetical protein (nitroreductase family protein)	1,71987852
SAV1129	SAUSA300_1028	isdB (sirH)	iron-regulated cell wall-anchored protein SirH	2,63602978
SAV1130	SAUSA300_1029	isdA	cell surface protein	1,74666548
SAV1131	SAUSA300_1030	isdC	hypothetical protein (heme transporter)	5,72529488
SAV1132	SAUSA300_1031	isdD	hypothetical protein (heme ABC transporter)	2,05986054
SAV1133	SAUSA300_1032	isdE (sirF)	putative iron transport lipoprotein SirF	3,22751395
SAV1134	SAUSA300_1033	isdF (sirG)	putative iron transport permease SirG	4,70998783
SAV1135	SAUSA300_1034	srtB	NPQTN specific sortase B	3,04069265
SAV1136	SAUSA300_1035	isdG	iron-regulated surface determinant G	2,18258827
SAV1350	SAUSA300_1246	citB	aconitate hydratase	1,27125784
SAV1414	SAUSA300_1307	arlS	putative protein histidine kinase	1,30997263
SAV1415	SAUSA300_1308	arlR	putative response regulator	1,99542124
SAV1731	SAUSA300_1677	isdH (harA)	hypothetical protein (heme-iron transporter)	0,7708115
SAV1893	SAUSA300_1874		ferritin	0,2118522
SAV2175	SAUSA300_2134	htsC	heme transport system permease	1,86379088
SAV2176	SAUSA300_2135	htsB	heme transport system permease	1,7519602
SAV2177	SAUSA300_2136	htsA	heme transport system lipoprotein	1,4607811
SAV2178	SAUSA300_2137		alanyl-racemase	2,70906185
SAV2179	SAUSA300_2138		FhuF-like transporter (aerobactin biosynthesis)	2,19300637
SAV2180	SAUSA300_2139		macrolide MFS transporter	3,42871185
SAV2181	SAUSA300_2140		FhuF-like transporter (aerobactin biosynthesis)	1,63623882
SAV2284	SAUSA300_2235	fhuD	ferric hydroxamate receptor 1 (substrate binding)	0,82431444
SAV2372	SAUSA300_2317	trxB	thioredoxin reductase	1,47880313
SAV2550	SAUSA300_2487	feoB	ferrous iron transport protein B	1,70050117
SAV2551	SAUSA300_2488	feoA	ferrous iron transport protein A	1,62796912

transcriptional unit (operon structure)

increase	> 2-fold	
	< 2-fold	
decrease	< 2-fold	
	> 2-fold	
